# Heterobinuclear Molecular Precursors Direct the Formation of Supported Subnanometer Cu–M Clusters with Tunable Catalytic Behavior

**DOI:** 10.1021/acsami.5c11995

**Published:** 2025-09-24

**Authors:** Mazal Kostan-Carmiel, Hadar Shema, Hsien-Cheng Yu, Griffin A. Canning, Dina Shpasser, Akshay Soni, Sergei Remennik, Neal Mankad, Robert M. Rioux, Oz Gazit, Elad Gross

**Affiliations:** † Institute of Chemistry, 26742 The Hebrew University, Jerusalem 91904, Israel; ‡ The Center for Nanoscience and Nanotechnology, The Hebrew University, Jerusalem 91904, Israel; § Department of Chemistry, 14681 University of Illinois at Chicago, 845 W. Taylor Street, Chicago, Illinois 60607, United States; ∥ Department of Chemical Engineering, 8082 The Pennsylvania State University, University Park, Pennsylvania 16802, United States; ⊥ Wolfson Faculty of Chemical Engineering, Technion-Israel Institute of Technology (IIT), Haifa 32000, Israel; # Department of Chemistry, The Pennsylvania State University, University Park, Pennsylvania 16802, United States

**Keywords:** bimetallic catalysts, subnanometer clusters, N-heterocyclic carbene, molecular precursors, ethylene hydrogenation

## Abstract

Subnanometer bimetallic clusters hold great promise for catalytic applications due to their unique electronic properties and high surface-to-volume ratios. However, precise control over their composition and size remains a major challenge, particularly for immiscible metal pairs. Here, we report a surface-anchored molecular approach for synthesizing ∼0.7–0.8 nm Cu–M (M = Ru, Mo, W, Fe) bimetallic clusters on mesoporous silica supports, using heterobinuclear N-heterocyclic carbene (NHC)-based complexes as precursors. The NHC ligand functionalized with an alkoxysilane anchor enables robust grafting to the silica interface. Controlled calcination and reduction lead to subnanometer clusters with tunable composition, dictated by the metal–metal bond stability in the precursor. In situ transmission electron microscopy reveals cluster growth proceeds via sintering of adjacent surface-bound units, while elevated temperatures above 300 °C triggering diffusion and phase separation. Catalytic testing in ethylene hydrogenation demonstrates composition-dependent activity and kinetics, with CuRu and CuW clusters exhibiting lower apparent activation energy barriers compared with monometallic Cu nanoparticles. This study establishes a generalizable strategy for the interfacial synthesis of alloyed clusters from molecular precursors and provides mechanistic insight into how precursor design governs nanostructure formation and catalytic behavior.

## Graphical abstract

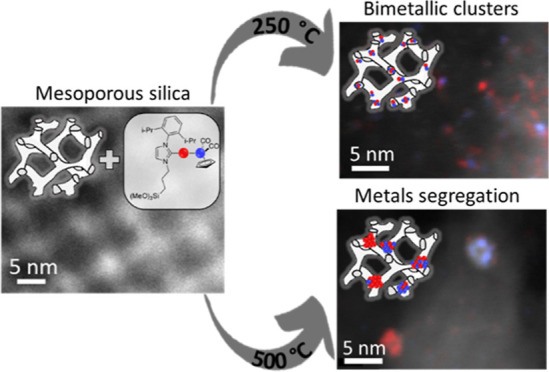

## 1 Introduction

Bimetallic nanoparticles play a significant role in catalytic processes^1–3^ as the combination of two metals can regulate the catalytic properties through geometric effects,^4^ electronic effects,^5^ lattice strain effects, and bifunctional effects.^6–11^ Bimetallic clusters in the subnanometer (<1 nm) range are anticipated to provide novel electronic and catalytic properties due to the resemblance of bimetallic clusters to the properties of highly reactive heterobinuclear organometallic complexes and their high surface-to-bulk ratio.^1,12–14^

Various synthetic strategies were envisioned to take advantage of the potential to alloy metals at the subnanometer scale, which are immiscible in the bulk.^15–18^ These syntheses would provide a novel route toward studying previously inaccessible bimetallic systems with unique electronic properties that cannot be attained with larger nanoparticles due to their propensity to follow bulk behavior and phase separation.^12,19–21^ Although the synthesis and enhanced reactivity of bimetallic nanoparticles has been widely demonstrated,^22–26^ synthesis of subnanometer bimetallic clusters is not trivial since it requires ultimate control over both size and composition.

Conventional impregnation methods, such as wet impregnation and deposition–precipitation, typically result in bimetallic nanoparticles with limited control over their size and composition, due to the lack of thermodynamic or kinetic control that regulates these properties.^29^ Therefore, various strategies for the synthesis of bimetallic nanoparticles with greater control over their composition and size have been developed, including colloidal synthesis^28–30^ and atomic layer deposition.^31^ These methods led to improved composition control but mostly induced the formation of nanoparticles at a size range of a few nanometers.

**Scheme 1:**
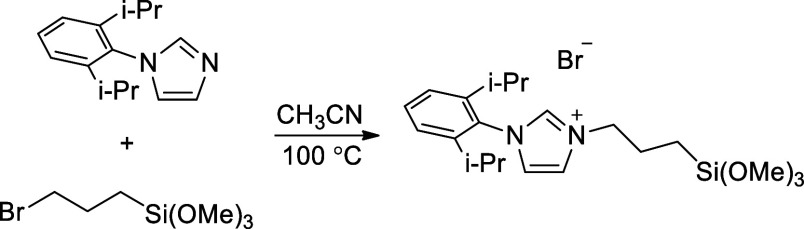
Preparation of *N*-(2,6-Diisopropylphenyl)-*N*′-(3-trimethoxysiloxyl-1-propyl)­imidazolium Bromide (L·HBr)

**Scheme 2:**
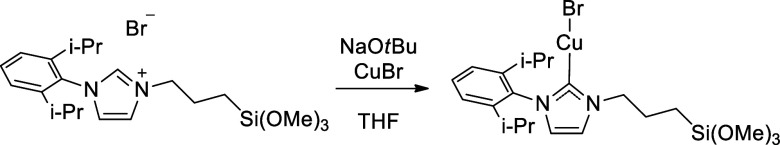
Preparation of LCuBr

**Scheme 3:**
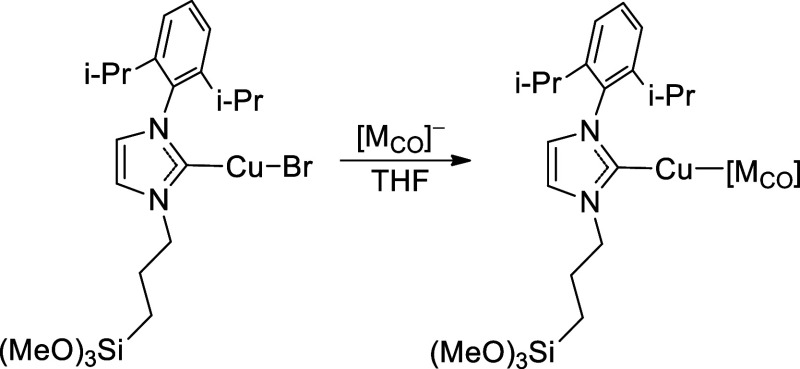
Preparation of L–Cu–M, M = FeCp­(CO)_2_, RuCp­(CO)_2_, MoCp­(CO)_3_, WCp*­(CO)_3_

**Scheme 4:**
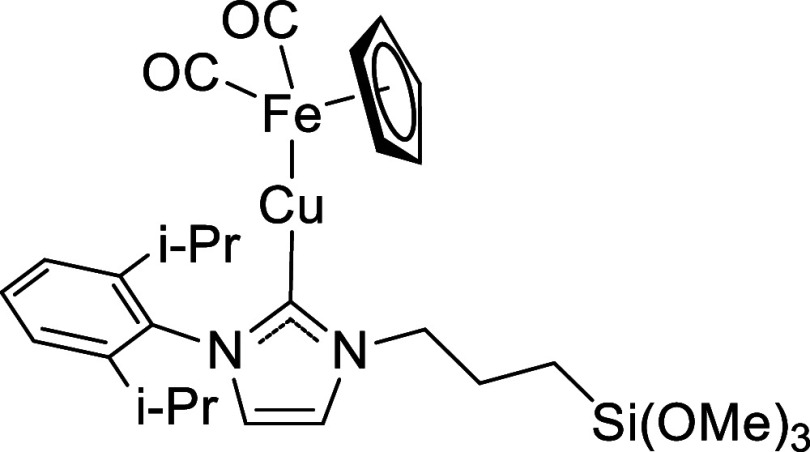
Chemical Structure of LCu-FeCp­(CO)_2_

**Scheme 5:**
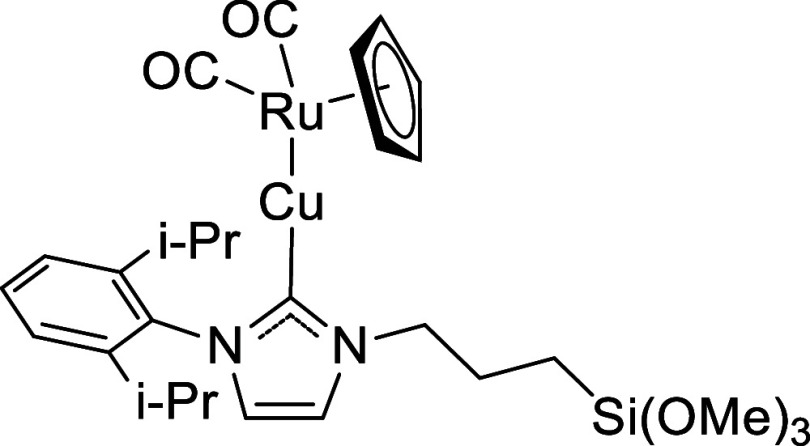
Chemical Structure of LCu-RuCp­(CO)_2_

**Scheme 6:**
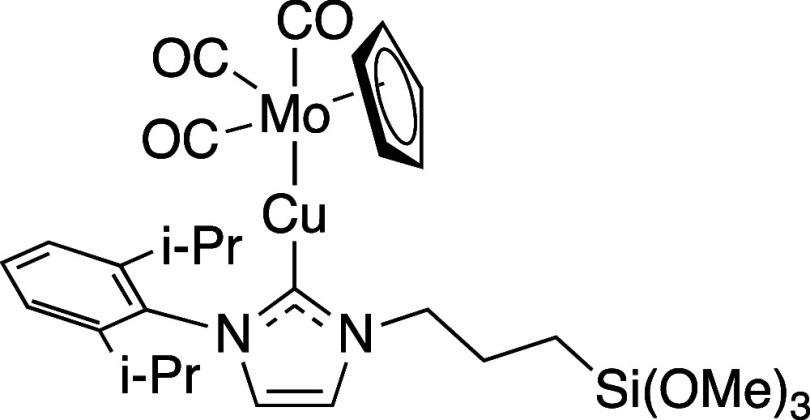
Chemical Structure of LCu-MoCp­(CO)_3_

**Scheme 7:**
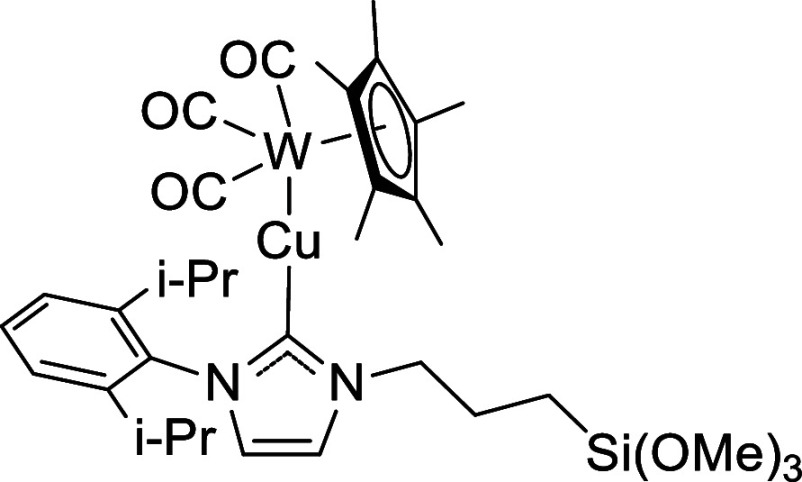
Chemical Structure of LCu-WCp*­(CO)_3_

Electrostatic interactions were utilized to guide the synthesis of bimetallic nanoparticles either by coadsorption of cationic metal complexes on negatively charged SiO_2_ or by sequential adsorption of oppositely charged metal complexes to form heterometallic double complex salts.^32,33^ Bimetallic nanoparticles in the size range of 1–3 nm were synthesized by these methods. However, the synthesis of subnanometer bimetallic clusters with controlled composition remains highly challenging.

Surface-grafted heterobinuclear complexes can ideally function as precursors for bimetallic cluster formation due to the intimacy and well-controlled atomic ratio of the two metals in the complexes.^34,35^ However, the instability of the grafted complexes hindered their utilization as precursors for bimetallic clusters formation. Monometallic organometallic or coordination complexes with a single metal atom were grafted on oxides and either used directly as single-atom catalysts or as precursors for the formation of monometallic nanoparticles following a thermal post-treatment.^36–43^ In addition, heterobinuclear and homobinuclear complexes with two different or identical metal atoms, respectively, were anchored on various surfaces and utilized as grafted molecular catalysts but were not utilized as precursors for bimetallic cluster formation.^44–49^

Herein, we demonstrate bimetallic complexes with N-heterocyclic carbene bearing an alkoxysilane anchor, which are characterized by strong metal–metal and metal–carbene bonds, for their utilization as precursors for the formation of bimetallic clusters. Heterobinuclear complexes were designed to enable control over the composition and size of bimetallic clusters based on two principles (Figure 1a). First, the metal atoms were coordinated to the surface via an NHC (*N*-heterocyclic carbene) ligand^50^ that was functionalized with an alkoxysilane group, thus affording both strong metal–ligand interactions and thermally stable surface grafting.^51^ Second, the two metals in the heterobimetallic complex form a strong metal–metal bond^52^ and were coordinated to a more labile carbonyl ligand.^53^ Due to these design principles, we were able to graft the heterobimetallic complexes on silica and, following thermal treatment, induce aggregation that enabled the formation of subnanometer Cu–M (M = Ru, Mo, Fe, W) clusters. These clusters demonstrated composition-dependent reactivity in ethylene hydrogenation, showing the crucial impact of cluster composition on the resulting reactivity.

**Figure 1:**
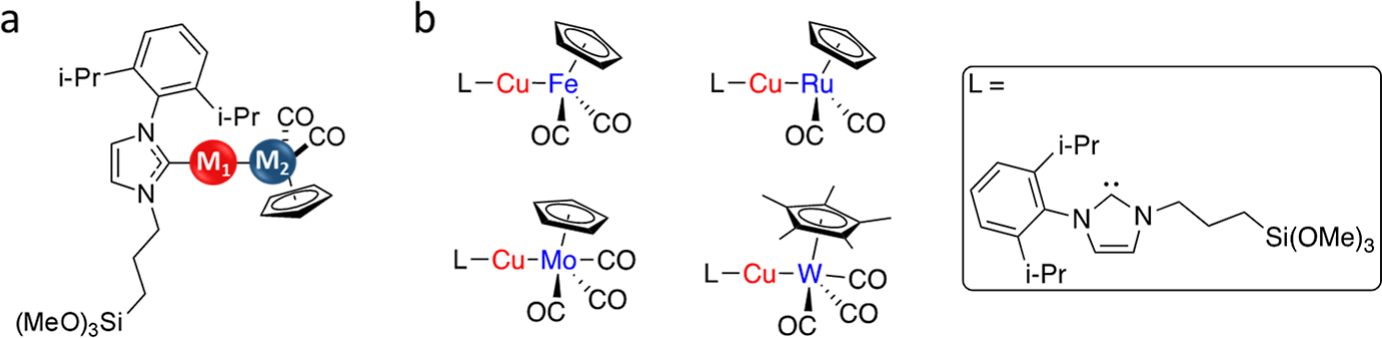
(a) Schematic illustration of the heterobinuclear complex (M_1_ = Cu, M_2_ = Ru, Mo, Fe, W). (b) Heterobinuclear complexes (L–Cu–M), which were utilized as precursors for bimetallic cluster formation.

## 2 Material and Methods

### 2.1 Synthetic Procedures

#### 2.1.1 L–Cu–M Synthesis General Considerations

Molecular synthesis procedures were carried out under a N_2_ atmosphere inside an MBraun Lab Master glovebox. All glassware was oven-dried before use. All reaction solvents were taken from a Glass Contour Solvent System built by Pure Process Technology, LLC, and further dried over molecular sieves in the glovebox. Deuterated solvents were degassed and stored over 3 Å molecular sieves. *N*-(2,6-Diisopropylphenyl)­imidazole was prepared using a literature procedure.^54^ The metal carbonyl salts K­[FeCp­(CO)_2_], Na­[RuCp­(CO)_2_], Na­[MoCp­(CO)_3_], and Na­[WCp*­(CO)_3_] were prepared using literature methods.^55–58^ All other reagents were purchased from commercial vendors and used without further purification. ^1^H NMR spectra were recorded by using Bruker AVANCE DPX-400 or DPX-500 MHz NMR spectrometers, and chemical shifts were referenced to the residual solvent peaks. FT-IR spectra were recorded using a Bruker ALPHA spectrometer fitted with a diamond-ATR detection unit.

#### 2.1.2 Preparation of *N*-(2,6-Diisopropylphenyl)-*N*′-(3-trimethoxysiloxyl-1-propyl)­imidazolium Bromide (L·HBr)

A literature procedure^59^ was modified as follows (Scheme 1). In a nitrogen-filled glovebox, a solution of *N*-(2,6-diisopropylphenyl)­imidazole (1.528 g, 6.50 mmol) and 3-(bromopropyl)­trimethoxysilane (1.368 g, 6.40 mmol) in acetonitrile (20 mL) was added to a 250 mL round-bottomed flask, which was sealed with a Teflon cap under an atmosphere of nitrogen. The mixture was heated to 100 °C for 12 h. After the mixture cooled to room temperature, the solvent was removed in vacuo, and the crude product (brown sticky residue) was triturated in diethyl ether (3 × 50 mL) to yield an off-white solid (2.11 g, 4.47 mmol, 79%). ^1^H NMR (500 MHz, CDCl_3_): δ 10.52 (s, 1H, NCHN), 7.73 (s, 1H, NCH), 7.54 (t, *J* = 7.5 Hz, 1H, *p*-H), 7.32 (m, 2H, *m*-H), 7.15 (s, 1H, NCH), 4.86 (t, *J* = 7.0 Hz, 2H, alkyl-CH_2_), 3.59 (s, 9H, –OCH_3_), 2.29 (sept, *J* = 7.0 Hz, 2H, **CH**(CH_3_)_2_), 2.13 (quint, *J* = 7.0 Hz, 2H, alkyl-CH_2_), 1.26 (d, *J* = 6.5 Hz, 6H, CH­(**CH**_**3**_)_2_), 1.15 (d, *J* = 6.5 Hz, 6H, CH­(**CH**_**3**_)_2_), 0.71 (t, *J* = 8.0 Hz, 2H, alkyl-CH_2_). Note: the completely dried imidazolium salt would display a different ^1^H NMR spectrum with many broad peaks. This issue can be resolved by redissolution in a solvent such as THF or Et_2_O, upon which the ^1^H NMR spectrum matches the literature data.

#### 2.1.3 Preparation of LCuBr

In a nitrogen-filled glovebox, L·HBr (504.0 mg, 1.29 mmol) was dissolved in THF (10 mL) in a 20 mL scintillation vial (Scheme 2). The solution was then transferred to a new 20 mL scintillation vial containing a mixture of CuBr (162.0 mg, 1.37 mmol) and NaO*t*Bu (132.0 mg, 1.54 mmol). The resulting yellow, cloudy solution was stirred for 12 h at room temperature. Then, the solution was pipet-filtered through Celite and dried in vacuo, resulting in a yellow-brown oily residue that was dissolved with diethyl ether (4 mL). This solution was evacuated to dryness again, giving a foamy semisolid gel. The residue was washed with a mixture of solvents (toluene/pentane = 1:4, 5 mL), which was decanted off to remove soluble impurities. The final product was dried in vacuo again to give yellow-brown oil (530.0 mg, 0.99 mmol, 77%). ^1^H NMR (500 MHz, CDCl_3_): δ 7.51 (t, *J* = 8.0 Hz, 1H, *p*-H), 7.31–7.24 (d, *J* = 9.0 Hz, 2H, *m*-H), 7.18 (s, 1H, NCH), 6.97 (s, 1H, NCH), 4.33 (t, *J* = 7.0 Hz, 2H, alkyl-CH_2_), 3.80 (br, s, 3H, –OCH_3_), 3.65 (s, 3H, –OCH_3_), 3.61 (s, 3H, –OCH_3_), 2.47–2.41 (m, 2H, **CH**(CH_3_)_2_), 2.12–2.07 (m, 2H, alkyl-CH_2_), 1.31 (d, *J* = 7.0 Hz, 6H, CH­(**CH**_**3**_)_2_), 1.17 (d, *J* = 7.0 Hz, 6H, CH­(**CH**_**3**_)_2_), 0.75–0.67 (m, 2H, alkyl-CH_2_).

#### 2.1.4 General Preparation of L–Cu–M

In a nitrogen-filled glovebox, LCuBr (1 equiv) was dissolved in THF (10 mL) in a 20 mL scintillation vial (Scheme 3). The solution was then transferred to a new 20 mL scintillation vial containing a Na­[M_CO_] or K­[M_CO_] reactant (1.1 equiv) and stirred for 12 h at room temperature, during which the solution changed color from yellow-brown to dark brown. After the reaction was completed, the solvent was dried in vacuo, giving a brown residue that was then extracted with a mixture of solvents (toluene/pentane = 1:2, 5 mL) through a Celite pipet. The filtrate was dried in vacuo again, resulting in a brown oil residue that was washed with pentane (4 mL) to remove the soluble impurities. The final product was dried in vacuo again to give a brown oil.

#### 2.1.5 Preparation of LCu-FeCp­(CO)_2_

The general procedure was followed with LCuBr (152.2 mg, 0.285 mmol) and Na­[FeCp­(CO)_2_] (63.3 mg, 0.316 mmol) (Scheme 4). Yield: 110.0 mg, 0.174 mmol, 61%. ^1^H NMR (500 MHz, C_6_D_6_): δ 7.21–7.18 (m, 1H, *p*-H), 7.07–7.03 (m, 2H, *m*-H), 6.15–6.03 (m, 2H, NCH), 4.34 (s, 5H, Cp), 3.86–2.82 (m, 1H, alkyl-CH_2_), 3.81–3.78 (m, 1H, alkyl-CH_2_), 3.56–3.54 (m, 3H, –OCH_3_), 3.45 (d, *J* = 5.0 Hz, 6H, –OCH_3_), 2.50 (sept, *J* = 8.5 Hz, 2H, **CH**(CH_3_)_2_), 1.91–1.83 (m, 2H, alkyl-CH_2_), 1.40–1.34 (m, 6H, CH­(**CH**_**3**_)_2_), 1.03–0.99 (m, 6H, CH­(**CH**_**3**_)_2_), 0.71–0.65 (m, 2H, alkyl-CH_2_). IR (solid, cm^–1^): 1908 (ν_CO_), 1840 (ν_CO_).

#### 2.1.6 Preparation of LCu-RuCp­(CO)_2_

The general procedure was followed with LCuBr (0.331 g, 0.619 mmol) and K­[RuCp­(CO)_2_] (0.183 g, 0.700 mmol) (Scheme 5). Yield: 0.30 g, 0.444 mmol, 71%. ^1^H NMR (500 MHz, C_6_D_6_): δ 7.22–7.19 (m, 1H, *p*-H), 7.09–7.05 (m, 2H, *m*-H), 6.13–6.03 (m, 2H, NCH), 4.85 (d, *J* = 7.5 Hz, 5H, Cp), 3.85–3.82 (m, 1H, alkyl-CH_2_), 3.82–3.78 (m, 1H, alkyl-CH_2_), 3.58–3.56 (m, 3H, –OCH_3_), 3.46 (d, *J* = 5.0 Hz, 6H, –OCH_3_), 2.50 (sept, *J* = 7.0 Hz, 2H, **CH**(CH_3_)_2_), 1.92–1.86 (m, 2H, alkyl-CH_2_), 1.42–1.37 (m, 6H, CH­(**CH**_**3**_)_2_), 1.05–1.01 (m, 6H, CH­(**CH**_**3**_)_2_), 0.73–0.68 (m, 2H, alkyl-CH_2_). IR (solid, cm^–1^): 1934 (ν_CO_), 1861 (ν_CO_).

#### 2.1.7 Preparation of LCu-MoCp­(CO)_3_

The general procedure was followed with LCuBr (0.252 g, 0.472 mmol) and Na­[MoCp­(CO)_3_] (0.141 g, 0.526 mmol) in Et_2_O/THF (2:1, 10 mL) (Scheme 6). Yield: 0.260 g, 0.372 mmol, 79%. ^1^H NMR (500 MHz, C_6_D_6_): δ 7.22 (t, *J* = 7.8 Hz, 1H, *p*-H), 7.08 (d, *J* = 7.7 Hz, 2H, *m*-H), 6.20 (m, 1H, NCH), 6.12 (s, 1H, NCH), 4.92 (s, 5H, Cp), 3.90 (m, 2H, alkyl-CH_2_), 3.57 (s, 3H, –OCH_3_), 3.47 (s, 6H, –OCH_3_), 2.52 (sept, *J* = 6.3 Hz, 2H, **CH**(CH_3_)_2_), 1.90 (m, 2H, alkyl-CH_2_), 1.38 (m, 6H, CH­(**CH**_**3**_)_2_), 0.99 (m, 6H, CH­(**CH**_**3**_)_2_), 0.65 (m, 2H, alkyl-CH_2_). IR (C_6_D_6_, cm^–1^): 1929 (ν_CO_), 1820 (sh, ν_CO_), 1795 (ν_CO_).

#### 2.1.8 Preparation of LCu-WCp*­(CO)_3_

The general procedure was followed with LCuBr (0.102 g, 0.191 mmol) and Li­[WCp*­(CO)_3_] (0.093 g, 0.227 mmol) in 8 mL of THF at 40 °C (Scheme 7). Yield: 0.111 g, 0.127 mmol, 66%. ^1^H NMR (500 MHz, C_6_D_6_): δ 7.26–7.23 (m, 2H, *m*-H), 7.14–7.11 (m, 1H, *p*-H), 6.24–6.13 (m, 2H, NCH), 4.05–4.02 (m, 1H, alkyl-CH_2_), 4.00–3.97 (m, 1H, alkyl-CH_2_), 3.59–3.57 (m, 3H, –OCH_3_), 3.48 (d, *J* = 5.0 Hz, 6H, –OCH_3_), 2.59 (sept, *J* = 7.0 Hz, 2H, **CH**(CH_3_)_2_), 2.01 (s, 15H, Cp*), 1.96–1.87 (m, 2H, alkyl-CH_2_), 1.47–1.43 (m, 6H, CH­(**CH**_**3**_)_2_), 1.03–0.98 (m, 6H, CH­(**CH**_**3**_)_2_), 0.71–0.67 (m, 2H, alkyl-CH_2_). IR (solid, cm^–1^): 1890 (ν_CO_), 1763 (ν_CO_).

#### 2.1.9 Synthesis of a KIT-6 Mesoporous Silica Support

KIT-6 was synthesized via a sol–gel process adapted from the literature.^60^ 9 g of Pluronic P123 were dissolved in 327 g of distilled water and 14.5 g of conc. HCl (35%). To this, 9 g of n-butanol was added under stirring at 35 °C. After 1 h of stirring, 19.3 g of tetraethoxysilane (TEOS) was added at 35 °C. The mixture was left stirring for 24 h at 35 °C and subsequently annealed for 24 h at 100 °C in the oven under static conditions in a closed polypropylene bottle. The solid product was filtered and dried at 100 °C. The template was removed by extraction in distilled water three times, followed by calcination at 550 °C.

#### 2.1.10 Preparation of Bimetallic Clusters

Mesoporous silica (KIT-6) was immersed and refluxed in water and then dried at 110 °C under vacuum.^61,62^ Afterward, the complex was immersed in dry toluene and added to the silica under an inert atmosphere. The mixture was stirred for 20 h. Following grafting, the silica was rinsed 3 times in toluene to remove physisorbed species, dried at RT under N_2_ flow and then stored in a desiccator for 24 h, and then annealed (100 °C, 1 h). The silica was then exposed to atmospheric conditions at a designated temperature (200–500 °C, 5 h) to remove solvent and organic residues, followed by reduction at 200–500 °C, 5 h, 1 atm of 5% H_2_, and 95% Ar.

#### 2.1.11 Synthesis of Copper Nanoparticles

Cu nanoparticles were prepared by deposition–precipitation on a silica (Davisil, grade 62) support; 190 mg of Cu­(NO_3_)_2_·3H_2_O (Sigma-Aldrich, 99–104%) and 4.75 g of urea were added to 188 mL of water heated to 80 °C and stirred at 100 rpm. After full dissolution of the urea, 1.00 g of silica was added. The reaction mixture was stirred for 4 h and then filtered and dried, followed by calcination (5 °C/min heating rate) in air at 300 °C for 2 h.

### 2.2 Bimetallic Complex and Cluster Characterization

Proton nuclear magnetic resonance (^1^H NMR) spectra were recorded on Bruker AVANCE DPX-400 MHz or Bruker AVANCE DRX-500 MHz spectrometers. FTIR spectra were recorded by using a Bruker ALPHA spectrometer fitted with an attenuated total reflectance (ATR) diamond detection unit. ATR Fourier transform infrared (ATR–FTIR) spectra were obtained with a Thermo Scientific Nicolet iS50 FTIR spectrometer, equipped with an iS50 ATR accessory (Thermo Fisher Scientific) and a diamond ATR module.

High-resolution scanning-transmission electron microscopy (HR-STEM) and elemental mapping using energy-dispersive X-ray spectroscopy (EDS) were performed using an aberration probe-corrected Themis Z G3 (Thermo Fisher Scientific) operated at 300 kV, equipped with an annular dark field detector (HAADF) from Fischione Instruments for STEM and a Super-X EDS detector (Thermo Fisher Scientific) for high collection efficiency elemental analysis. Image and EDS elemental map analyses were performed with Velox (Thermo Fisher Scientific).

Thermogravimetric analysis mass spectroscopy (TGA–MS) measurements were performed using a SETARAM Labsys-Evo coupled to a Hiden QGA-pro using synthetic air as the carrier gas (20% O_2_ and 80% Ar_2_, 30 mL min^–1^, 5 °C min^–1^). All samples were annealed to 120 °C prior to TGA–MS measurements in order to remove physisorbed and solvent residues.

Diffuse reflectance infrared Fourier transform (DRIFT) measurements were performed using a custom setup based on an FTIR instrument (Bruker VERTEX 70v), with DRIFT setup (Harrick) and mass flow controllers (MKS). 5 wt % Cu–M complex was mixed with KBr and fixed in the DRIFT setup. DRIFT measurements were performed under a gas flow of 20% O_2_ and 80% N_2_.

Inductively coupled plasma optical emission spectroscopy (ICP–OES) measurements (Agilent 715) were performed by dissolving the sample in 4 mL of aqua regia, followed by dilution with deionized water. Samples were further diluted to maintain final concentrations of metals between 1 and 5 ppm.

X-ray photoelectron spectroscopy (XPS) measurements were performed by using a Kratos AXIS Ultra instrument equipped with a focused monochromatic Al Kα X-ray source (1486.7 eV). The X-ray beam was normal to the sample, and the photoelectron detector was positioned at 45° off-normal. C 1s (binding energy = 284.5 eV) was used as a reference for correction of any charging effects.

### 2.3 Reactivity Measurements

Reaction rate measurements were carried out in a plug flow reactor under a total pressure of 1.1 bar (controlled by a back-pressure regulator). Catalysts were reduced for 1 h at 250 °C before kinetic measurements and for 1 h at 250 °C before each subsequent measurement of rates under new reaction conditions (i.e., bracketing). Reaction products were analyzed using a Shimadzu 2010 QP Ultra gas chromatography mass spectrometry (GC–MS) instrument equipped with a Restek AluminaBond/KCl 30 m capillary column and a flame-ionization detector. Gas space velocity was varied between 0.71 and 0.14 mol ethylene g^–1^ catalyst h^–1^ to maintain differential conversion. Total gas flow was 96,000 mL gas h^–1^ g^–1^ catalyst bed. Apparent activation energy was measured between 40 and 65 °C with a H_2_ partial pressure of 29.3 kPa, ethylene partial pressure of 0.7 kPa (40:1 molar ratio), and balance helium. For the measurement of H_2_ order, ethylene pressure was fixed at 0.7 kPa and the H_2_ pressure was varied between 14 and 73 kPa. For measurement of the ethylene order, hydrogen pressure was fixed at 29.3 kPa and ethylene pressure was varied from 0.4–1.9 kPa. Reaction orders in both ethylene and H_2_ were measured at 75 °C.

## 3 Results and Discussion

### 3.1 Design and Surface Anchoring of Heterobinuclear Complexes

Heterobinuclear complexes (L–Cu–M) were employed as precursors to mediate the synthesis of bimetallic clusters in mesoporous silica.^50,52,56,63^ To access the L–Cu–M (M = Ru, W, Mo, Fe) series shown in Figure 1, the LCuBr precursor reacted with a metal carbonyl anion, [M]^−^ (e.g., Na­[FeCp­(CO)_2_] in which Cp = η^5^-C_5_H_5_), to form the (NHC)­Cu–M complex driven by salt precipitation (M = RuCp­(CO)_2_, WCp­(CO)_3_, MoCp­(CO)_3_, and FeCp­(CO)_2_).^20,21,25^ Immobilization of NHC ligands onto silica supports was achieved using ligand L (Figure 1b) as a surface anchoring group.^27,59^ All complexes were isolated as oils or oily semisolids and characterized by ^1^H NMR and FTIR measurements (Figure S1–S8).

L–Cu–M complexes were grafted onto mesoporous silica (KIT-6). To enhance the surface density of hydroxyl groups, which function as binding sites, the mesoporous silica was first immersed and refluxed in water^61^ and then annealed at 110 °C under vacuum.^62^ The grafted L–Cu–M complexes were characterized by diffuse reflectance infrared Fourier transform spectroscopy (DRIFTS), inductively coupled plasma optical emission spectroscopy (ICP–OES), and X-ray photoelectron spectroscopy (XPS) measurements (Figures S9 and S10). IR measurements probed the CO vibrations of the carbonyls (Figure S11). The CO vibration frequency of the surface-anchored complexes shifted by up to 86 ± 44 cm^–1^ in comparison with the precursor. The shifts in the CO stretching peak can be attributed to several factors, including interactions between the metal center and oxygen atoms on the support, loss of coordinated Cp or carbonyl ligands, and changes in the geometry of the carbonyl groups.^28,64,65^ Additionally, dissociation of the metal–metal (M–M) bond can alter the character of the metal–carbonyl (M–CO) bond, which can induce shifts in the CO vibrational spectra.

The surface density of the grafted complexes was found to range between 0.001 and 0.011 complexes·nm^–2^ (Table S1 in the Supporting Information). These values are 1 to 2 orders of magnitude lower than those previously reported for grafted complexes.^66–68^ The lower surface density is likely due to the larger molecular size of the grafted complexes, which limits their diffusion within the mesoporous framework and decreases the probability of forming chemical bonds with the silica surface.

XPS analysis of the precursors before and after grafting showed relatively minor changes in the Cu 2p signal, while broadening and shifts were observed in the XPS signal of the second metal (Figure S9 and S10). These results indicate that the second metal center (M) interacts more strongly with the surface, which may lead to the dissociation of the Cu–M bond. The variance in the surface density of the complexes was also identified by XPS analysis of the atomic percentage of the complexes, with a higher atomic percentage of CuFe and CuMo (Table S2). However, as will be detailed below, the higher surface density is mostly the result of physisorbed species that were not anchored to the surface, as identified by the decrease in the overall atomic percentage of CuFe and CuMo following exposure to calcination and reduction. A thorough spectroscopic analysis of cluster formation mechanism, and of the factors underlying variations in cluster composition and density, will be addressed in the following sections of this paper (Section 3.3).

### 3.2 Thermal Transformation and Structural Evolution of Bimetallic Clusters

Cu–M clusters were prepared by calcination of the grafted heterobimetallic complexes (in air under 1 atm), followed by reduction (5% H_2_ and 95% N_2_ at 1 atm) at 250 °C (Figure 2). High-angle annular dark-field scanning transmission electron microscopy (HAADF-STEM) images of the resulting Cu–M clusters and their size distribution are shown in Figure 3a and b, respectively, revealing clusters in the size range of 0.7–0.8 nm with a relatively narrow size distribution. The optimized protocol of calcination and reduction was developed since the direct reduction of the sample, without calcination, led to the formation of larger nanoparticles with wider size distribution (Figure S12). This is attributed to the exothermic process of ligand removal under reducing environment.

**Figure 2:**
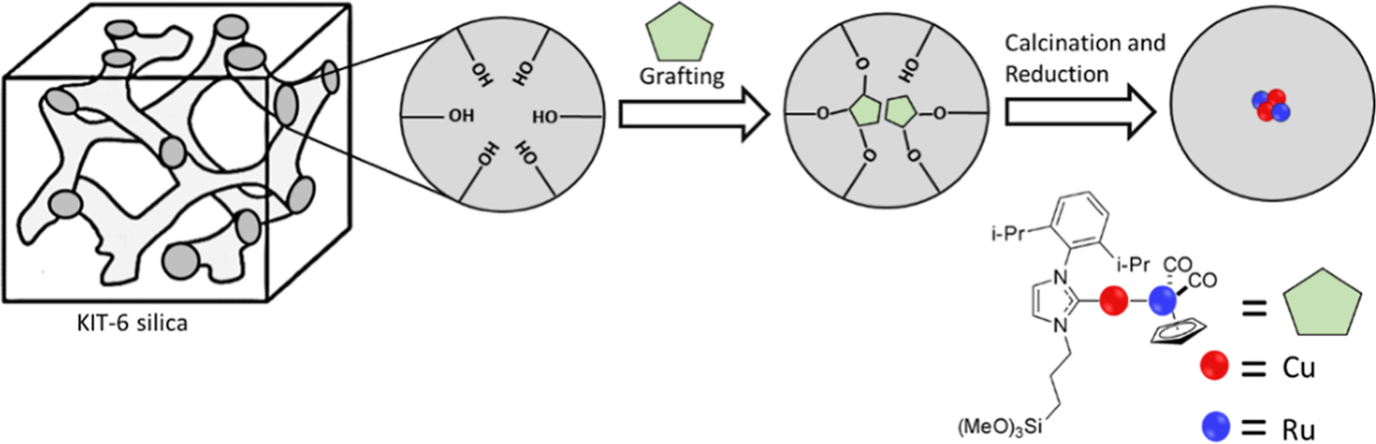
Schematic illustration depicts the approach for bimetallic cluster formation. For example, LCuRuCp­(CO)_2_ was grafted on a KIT-6 support, followed by calcination and exposure to reducing conditions.

**Figure 3:**
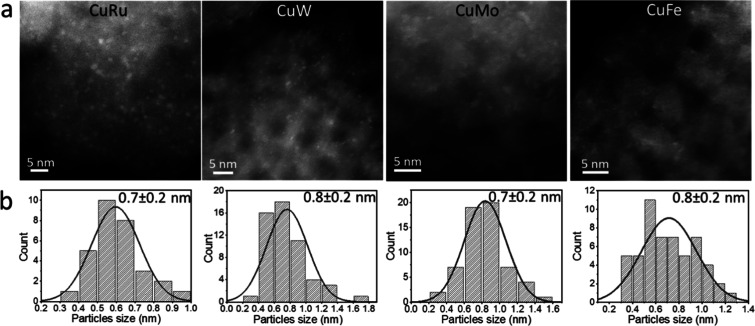
(a) HAADF-STEM images of Cu–M clusters where M corresponds to Ru, W, Mo, and Fe from left to right, respectively. (b) Size distribution analysis of the clusters shown in a.

STEM energy-dispersive X-ray spectroscopy (EDS) measurements were performed to assess the average composition of bimetallic clusters by examining the single particle composition of >100 clusters for each bimetallic system (Table 1, Figures S13–S19). ICP–OES measurements were performed to quantify the weight loading of the two metals in the silica and their ratios (Table 1). Higher weight loading and an approximate 1:1 ratio of the two metals were measured for Cu–Fe and Cu–Mo, while lower weight loading and higher Cu/M ratio were measured for CuRu and CuW.

**Table 1:** Weight and Composition Analysis of Cu–M Clusters

			Cu/M atomic ratio	
Cu–M sample	Cu wt %	M wt %	STEM–EDS	ICP–OES
CuFe/KIT-6	0.17 ± 0.01	0.19 ± 0.01	0.9 ± 0.4	0.8 ± 0.1
CuMo/KIT-6	0.27 ± 0.01	0.41 ± 0.01	0.6 ± 0.2	1.0 ± 0.1
CuRu/KIT-6	0.04 ± 0.01	0.02 ± 0.01	1.4 ± 0.3	3.1 ± 0.2
CuW/KIT-6	0.06 ± 0.01	0.11 ± 0.01	1.9 ± 0.3	1.5 ± 0.2

The higher than expected Cu/M ratio in CuRu and CuW is indicative of partial M–M bond cleavage during the anchoring stage, inducing a higher Cu/M ratio since Cu is coordinated to the surface-coordinated group. The presence of dissociated RuCp­(CO)_2_, WCp­(CO)_3_ physisorbed species can block adsorption sites and thus mitigate the chemisorption of LCuRu and LCuW complexes, respectively, and lead to a lower overall weight loading. These results show the crucial impact of M–M bond stability on the surface density and composition of the resulting bimetallic clusters. The higher Cu/M ratio observed in ICP–OES measurements compared to STEM–EDS for most complexes is attributed to the limited sensitivity of STEM–EDS in detecting atomic clusters and molecular fragments residing on the surface. Cu/M values greater than one are attributed to M–M bond cleavage, which leads to an increased relative Cu content, as Cu is directly coordinated to the silica surface.

Calcination followed by reduction led to aggregation and formation of subnanometer clusters, as demonstrated for Cu–Ru and Cu–Fe in the HAADF-STEM images shown in Figure 4 and for the other bimetallic systems in Figure S29. The average size of the clusters increased from subnanometer to >1 nm following exposure to higher calcination and reduction temperatures, as summarized in Table 2. Exposure of the sample to calcination and a reduction temperature of up to 500 °C further increased the average size of the clusters and their size distribution (Figure 4). STEM–EDS analysis revealed metal segregation within single particles occurred once the calcination and reduction temperature was >300 °C (Table S3 and Figures S20–S29). This observation shows that in larger nanoparticles, the bulk immiscibility leads to phase separation of the two metals. Consideration should be given to the fact that accurate acquisition of the composition of single clusters was limited due to the instability of the sample under prolonged exposure to the electron beam (Figure S30). Thus, the composition analysis in STEM–EDS measurements was based on an averaged analysis of the metal signals on several different regions in order to gain a better and more valid statistics.

**Figure 4:**
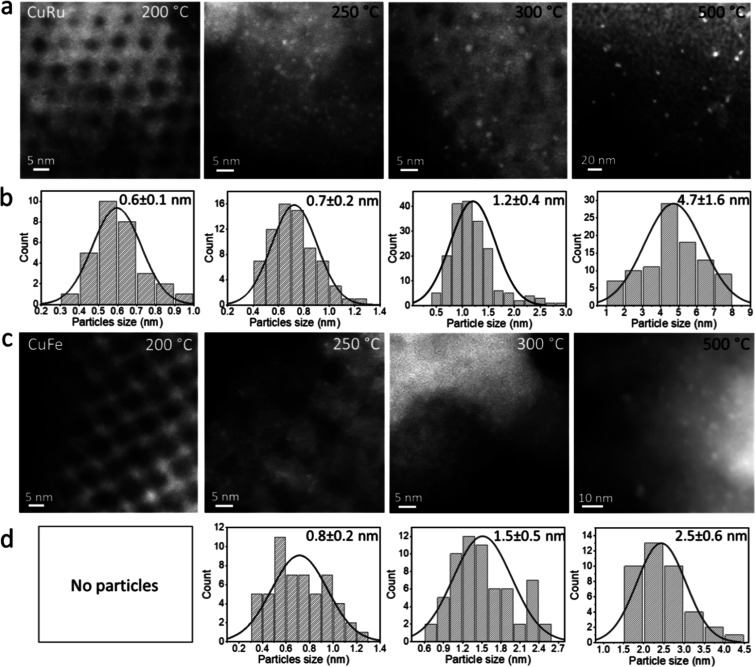
HAADF-STEM images (a,c) and the corresponding particles’ size distribution (b,d) of CuRu (a,b) and CuFe (c,d) clusters prepared by calcination and reduction at 200, 250, 300, and 500 °C.

**Table 2:** Average Size of Cu–M Clusters after Calcination and Reduction

	NP size (nm)	
Cu–M sample	250 °C	300 °C
CuRu	0.7 ± 0.2	1.2 ± 0.4
CuFe	0.8 ± 0.2	1.5 ± 0.5
CuMo	0.7 ± 0.2	1.0 ± 0.2
CuW	0.8 ± 0.2	1.6 ± 0.4

XPS analysis of the atomic percentages of the two metals after grafting and following calcination and reduction (Table S2) revealed that the metal density and Cu/M ratio in CuRu and CuW remained unchanged upon these treatments. In contrast, the overall metal content in both CuFe and CuMo decreased after calcination and reduction, indicating that physisorbed species were removed from the surface during the process. The XPS measurements exhibited a trend in the Cu/M ratio similar to the STEM–EDS analysis (Table 1), further confirming that the averaged STEM–EDS measurements provide a reliable estimation of the clusters’ composition.

*In situ* TEM measurements of grafted Cu–Ru complexes were conducted under a variable temperature to elucidate the growth pattern of bimetallic clusters (Figure 5). TEM measurements did not probe any particles following annealing to 100 °C (Figure S31), while clusters with an average size of 0.7 ± 0.2 nm were detected following annealing at 200 °C (Figure 5a). The averaged cluster size increased to 0.8 and 1.0 nm after short annealing periods to 250 and 300 °C, respectively, which were conducted in the TEM setup under ultra-high vacuum conditions. Quantitative EDS analysis at 300 °C revealed a Cu/Ru ratio of 2:1 (Figure S31), which is similar to the results described in Table 1. Analysis of the particles’ growth pattern was identified by continuously monitoring the changes in particle’s size on the same region while increasing the annealing temperature. Larger particles were formed either via coalescence of two smaller particles (Figure 5c) or by atomic migration (Figure 5d), inducing a wider particle size distribution.

**Figure 5:**
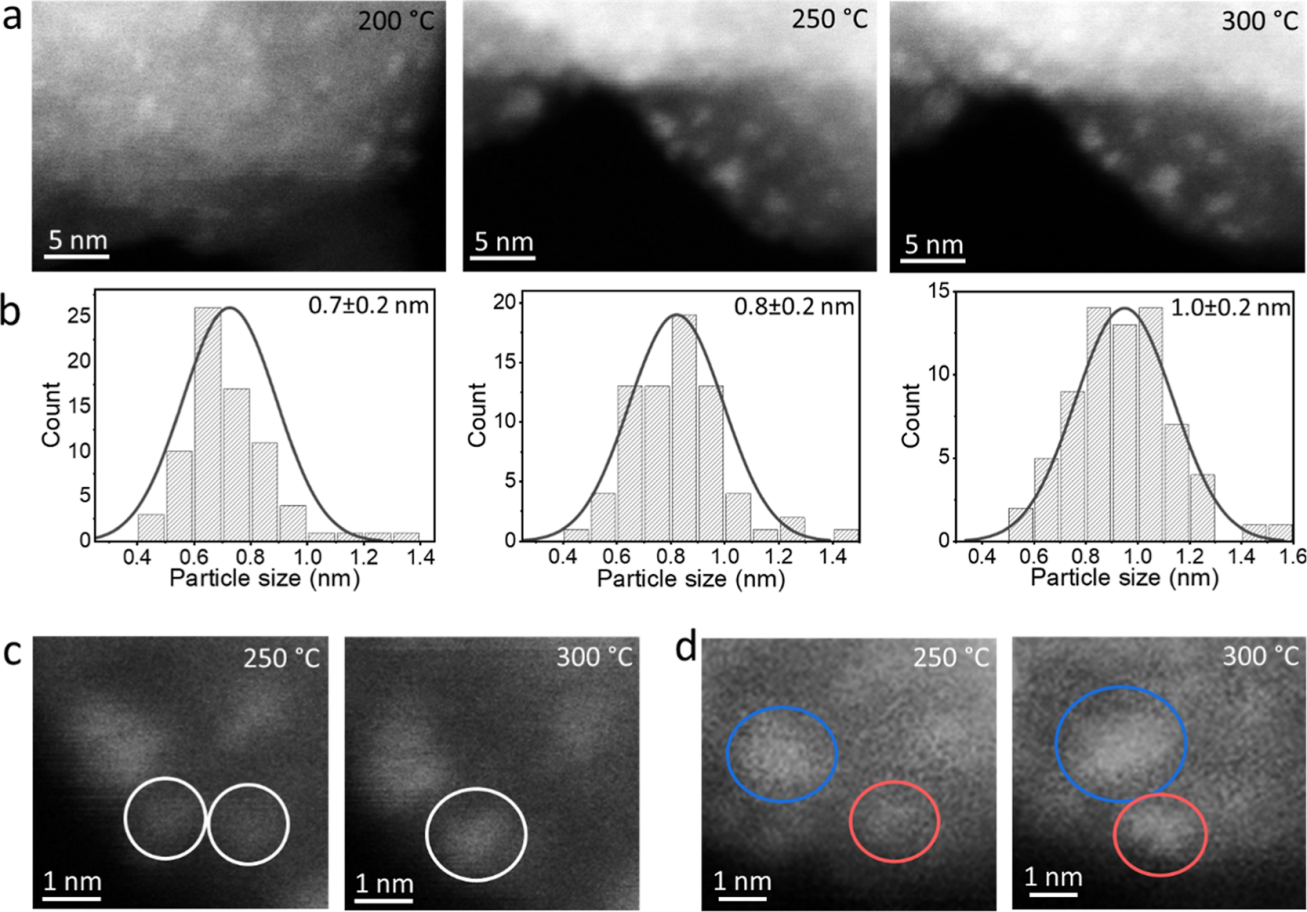
In situ HAADF-STEM imaging (a) and the corresponding particle size distribution (b) of grafted Cu–Ru complexes on silica under annealing to 200, 250, and 300 °C. HAADF-STEM images of the same regions under varying temperatures are shown in c and d and demonstrate particle growth via sintering of two subnanometer clusters (c) or sintering of smaller species (d).

The microscopic and spectroscopic data therefore indicate that Cu–Mo and Cu–Fe show higher stability during the grafting stage, which led to higher weight loading and an approximate 1:1 ratio of the two metals. Lower stability of the metal–metal bond in Cu–Ru and Cu–W led to a lower weight loading and higher Cu/M ratio. Annealing to 250 °C induced sintering and formation of subnanometer clusters, while annealing to higher temperatures led to formation of nanometer-sized particles. Additional TEM images are included in the Supporting Information (Figure S32) and further validate that cluster formation is induced by aggregation of neighboring clusters and by diffusion and aggregation of smaller atomic species, which cannot be resolved in the TEM imaging due to their small size.

### 3.3 Spectroscopic Analysis of Cluster Formation Mechanism

Thermogravimetric analysis combined with mass spectrometry (TGA–MS) measurements was performed under 1 atm of 20% O_2_ and 80% Ar to identify ligand dissociation and desorption and its correlation with bimetallic cluster formation. TGA measurements of the grafted Cu–Mo and Cu–Fe complexes identified five dominant mass loss peaks at 110, 250, 320, 450, and 550 °C (Figure 6a,c). The stepwise temperature ramp was designed to resolve overlapping mass loss events and to allow for accurate correlation between mass spectrometry signals and mass loss profiles. The low-temperature desorption peak at 110 °C was correlated to water desorption as identified by mass loss analysis of the KIT-6 substrate (Figures S33–S35). Mass loss at 250 °C was coupled with an increase in the CO_2_ and NO signatures (red and blue colored, respectively, Figure 6b,d), which can be correlated to carbonyl desorption, coupled with partial dissociation of the carbene ligand.

**Figure 6:**
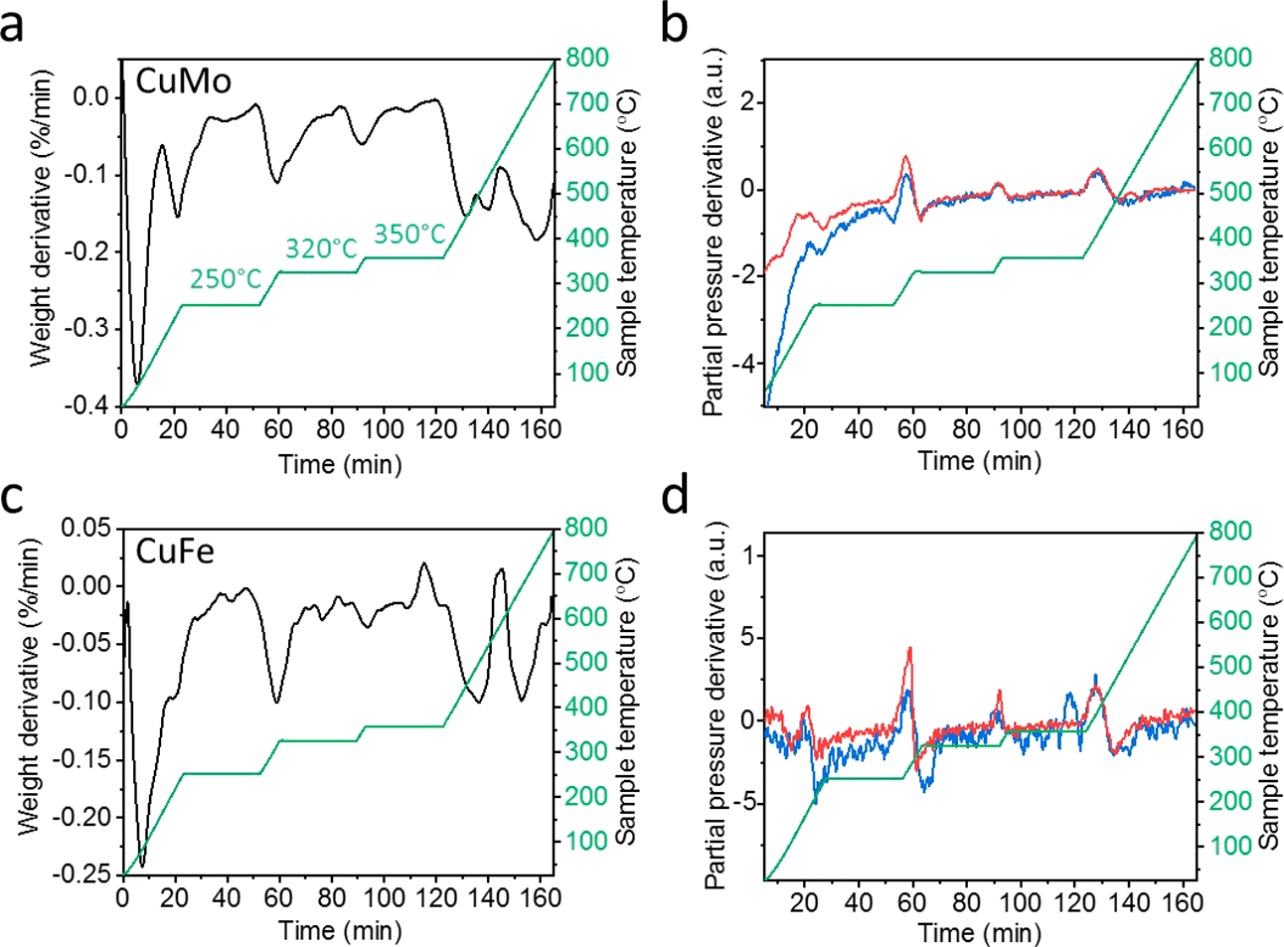
TGA–MS measurement analysis of grafted LCuMoCp­(CO)_3_ (a,b) and LCuFeCp­(CO)_2_ (c,d) complexes. (a) and (c) display the TGA mass loss derivatives, while panels (b) and (d) are the corresponding MS derivatives of CO_2_ (red) and NO (blue) signals. The NO signal was amplified by 25 for LCuMoCp­(CO)_3_ and 20 for LCuFeCp­(CO)_2_.

Dominant mass loss signals were detected at 320 °C (Figure 6a,c), which was coupled with the detection of noticeable CO_2_ and NO signals (Figure 6b,d). The noticeable detection of the CO_2_ and NO signals was correlated to dissociation and desorption of the carbene moiety, which is connected to the surface anchoring group. It is hypothesized that the dissociation of the surface-anchored carbene ligand at elevated temperatures enabled metal diffusion and the formation of larger nanoparticles by sintering of smaller clusters, as obtained by HR-TEM imaging (Figure 5). Mass loss signatures at 450 °C and above were correlated to desorption of chemisorbed fragments, since CO_2_ and carbon-based ligands are desorbed at lower temperatures.^69^

Temperature-dependent N 1s XPS measurements were performed on the grafted complexes to probe the carbene ligand dissociation and desorption pattern following exposure to elevated temperatures (Figure 7). For all samples, the N 1s XPS signal showed a peak at 400.5 eV, which was assigned to the N–C signature of carbene.^70–72^ The C 1s XPS signal was constructed of several Gaussians, assigned to C–C signatures, and higher binding energy components attributed to oxidized carbon species, such as C–O and C=O (Figure S36).

**Figure 7:**
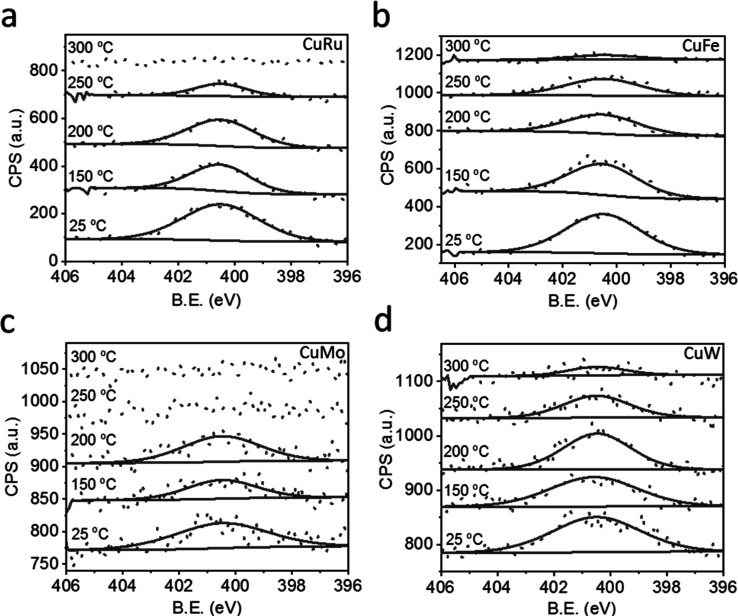
Temperature-dependent N 1s XPS measurements of grafted (a) Cu–Ru, (b) Cu–Fe, (c) Cu–Mo, and (d) Cu–W complexes before and after their calcination at 150, 200, 250, and 300 °C.

A continuous decrease in the amplitude of the N 1s XPS signature was detected upon annealing. The N 1s XPS signal of Cu–Mo and Cu–Ru disappeared after annealing to 250 and 300 °C, respectively. For the Cu–Fe and Cu–W systems, the signal was reduced to the noise level after annealing to 300 °C. The XPS results therefore indicate that carbene ligand decomposition and desorption occurred after annealing to 250–300 °C. These results further validate the formation of subnanometer clusters by annealing at >250 °C, as the restricted mobility of the metal atoms, induced by surface anchoring, prevented their excessive growth. At temperatures higher than 300 °C, the decomposition of the surface-anchored carbene ligands allowed metal diffusion, leading to particle growth in the nanometer range. Carbonyl desorption from the grafted complexes at 250 °C was identified by DRIFT measurements (Figure S37).

Integration of the spectroscopic data indicates that exposure of the complexes to a calcination temperature of ∼250 °C results in carbonyl desorption and partial decomposition of the carbene ligands which enabled the formation of subnanometer clusters. It is hypothesized that these clusters were formed by sintering of neighboring complexes or atomic clusters. Higher temperatures provided sufficient thermal energy for ligand decomposition and sintering of subnanometer clusters into larger (>1 nm) nanoparticles.

### 3.4 Catalytic Reactivity Measurements

The catalytic reactivity of Cu–Ru, Cu–Mo, Cu–W, and Cu–Fe clusters toward ethylene hydrogenation was measured to assess the impact of composition on reactivity (Figures 8a). Cu–Ru, Cu–Mo, Cu–W, and Cu–Fe clusters in the size range of 0.7–0.8 nm were prepared by calcination and reduction at 250 °C, before the reactivity measurements. All ethylene conversions were kept below 10%, allowing to assume a differential reactor and to calculate reaction rates as a function of temperature or partial pressure of reactants. The apparent activation energy values for Cu–Ru, Cu–Mo, Cu–W, and Cu–Fe were 45, 86, 45, and 65 kJ/mol, respectively. The large variation in the activation energy values demonstrates the crucial role of the alloying metal on reactivity. The reported activation energy values for ethylene hydrogenation over Cu alloy particles (such as Cu–Ni and Cu–Pd alloys) range from 38 to 50 kJ/mol, with specific values depending on the alloy composition and experimental conditions.^73,74^ The values reported for Cu–Ru and Cu–W systems fall within this range and are lower than those typically observed for monometallic Cu nanoparticles. The higher activation energy measured for Cu–Mo may indicate a lower binding energy of ethylene to this bimetallic system.

**Figure 8:**
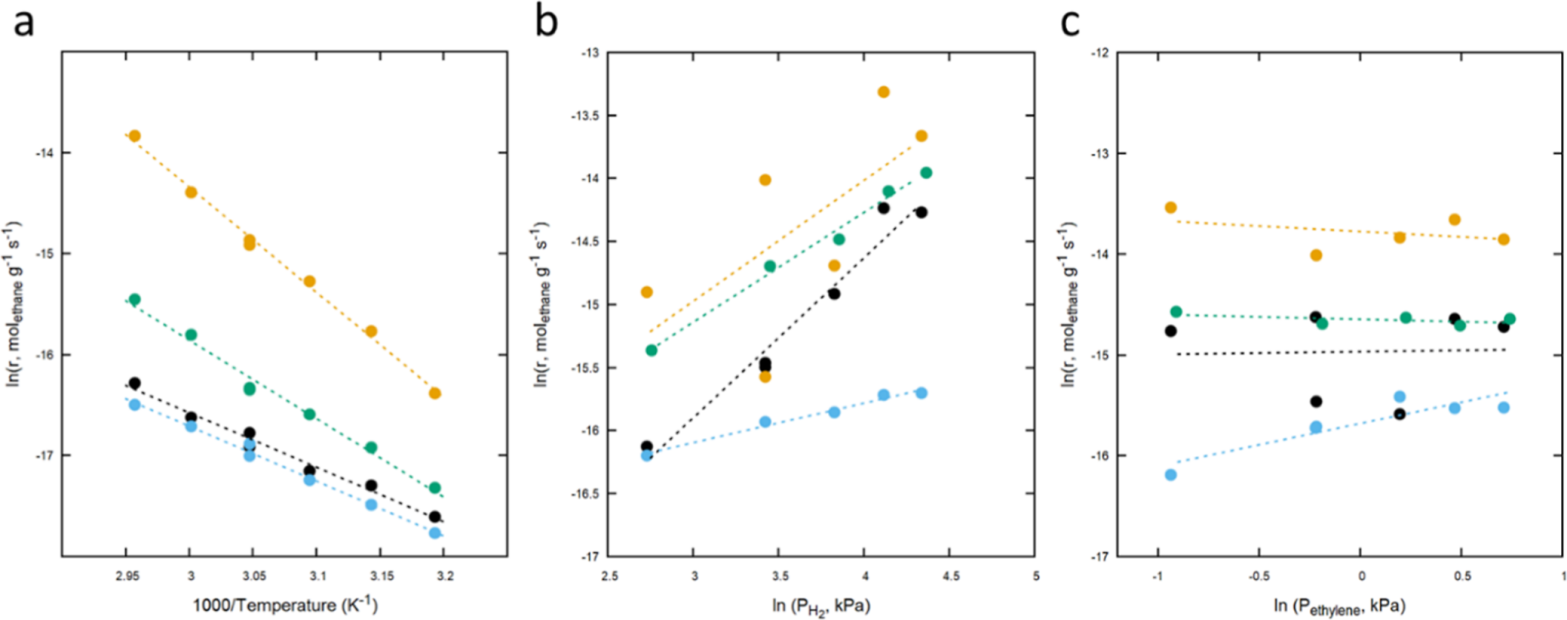
(a) Ethylene hydrogenation reaction rates as a function of temperature for Cu–Ru (black), Cu–Mo (gold), Cu–W (blue), and Cu–Fe (green). Reactions were conducted at 29.3 kPa hydrogen and 0.7 kPa ethylene at a total pressure of 111 kPa (balance helium). The gas hourly space velocity was held at 96 L g^–1^ catalyst h^–1^. (b) The rate of ethylene hydrogenation as a function of hydrogen pressure at 75 °C. Ethylene pressure was held constant at 0.7 kPa, while hydrogen pressure was varied over 14–73 kPa. The gas hourly space velocity was held at 96 L g^–1^ catalyst h^–1^. (c) Dependence of the rate of ethane formation on ethylene partial pressure at 75 °C. Hydrogen partial pressure was held at 29.3 kPa, while ethylene pressure was varied over 0.4–1.9 kPa. Gas hourly space velocity was 96 L g^–1^ catalyst h^–1^.

The reactivity of a monometallic copper catalyst supported on silica was measured for comparison (Figures S38–S40). The measured apparent activation energy for the Cu nanoparticles was 55 kJ mol^–1^. Both Cu–Ru and Cu–W yielded lower activation energy values than the supported Cu nanoparticles, although the Cu concentration in these clusters was more dominant than that of Ru and W (Table 1). Comparison of the reactivity of bimetallic clusters to Cu nanoparticles demonstrates that the alloyed clusters induce different reactivity patterns in comparison to monometallic Cu, dependent upon the combination of the two metals.

Additional kinetic measurements were conducted to gain a better understanding of the impact of the alloying metal on the reaction mechanism. Reaction orders were measured for hydrogen (Figure 8b) and ethylene (Figure 8c). The reaction order for hydrogen was 1.3, 1.0, 0.3, and 0.9 for Cu–Ru, Cu–Mo, Cu–W, and Cu–Fe, respectively. Reaction order for ethylene was 0.0, −0.1, 0.4, and 0.0 for Cu–Ru, Cu–Mo, Cu–W, and Cu–Fe, respectively. Reaction rates of Cu–Ru, Cu–Mo, and Cu–Fe displayed a strong dependence on hydrogen pressure but were nearly independent of ethylene pressure. Conversely, the rates of Cu–W showed intermediate dependence on both hydrogen and ethylene.

The Cu–Mo catalyst demonstrated instability during the hydrogen reaction order measurement, with evidence for changes in activity with the repeat data point. Interestingly, the same catalyst was stable during apparent activation energy and ethylene reaction order experiments. The Cu–Ru catalyst was stable during apparent activation energy and hydrogen reaction order measurements but demonstrated instability during ethylene reactor order measurements. However, four out of six rates measured at varying ethylene partial pressures clearly demonstrate zero order in ethylene behavior. We decided not to investigate this behavior any further since the emphasis of this work is to demonstrate the reactivity of subnanometer clusters and the consequence of M metal identity on observed ethylene hydrogenation kinetics.

The kinetics of ethylene hydrogenation over a monometallic copper catalyst supported on silica was measured for comparison. The reaction orders were 0.9 in hydrogen and 0.0 in ethylene, which were in good agreement with literature values (Table S4). No direct correlation was identified between the kinetic behavior of the alloying metal in its monometallic form^75–79^ and the results observed on the bimetallic particles, which demonstrate the effectiveness of Cu in impacting the reaction mechanism.

Reaction orders on Cu–Mo and Cu–Fe are generally consistent with reports on monometallic copper catalysts, suggesting catalysis by these particles may be dominated by surface ensembles that locally resemble copper or that the alloying metal has a weak effect on the reaction mechanism. The order above 1 in hydrogen for the Cu–Ru catalyst suggests hydrogen is responsible for generating the active state of the catalyst, for example, by lowering the surface coverage of ethylidyne or acetylide species, which block surface sites.^80,81^ Inclusion of copper into ruthenium appears to increase ethylene binding strength on ruthenium, which could lead to enhanced formation rate of the reactive species.^75^ Cu–W displays reaction orders that are positive in both ethylene and hydrogen; this can be explained by either one of the C–H bond formation(s) as the rate-determining step but is typical of systems with relatively weaker ethylene binding energy. These effects also result in a lower activation energy barrier. It is identified that the formation of bimetallic Cu–Ru and Cu–W clusters without phase separation modified the reaction mechanism and induced noticeable changes in their catalytic properties.

Long-term stability was measured (>15 h) under reaction conditions and did not reveal a rapid deactivation and indicates that no abrupt changes in the catalyst’s structure were induced under reaction conditions (Figure S41). TEM measurements of the spent catalyst showed minor changes in clusters’ size after exposure to reaction conditions, demonstrating the high stability of the clusters during the reaction (Figure S42).

## 4 Conclusions

We demonstrate the synthesis of subnanometer bimetallic Cu–M (M = Ru, Mo, Fe, W) clusters using surface-grafted heterobinuclear complexes as precursors. The N-heterocyclic-carbene/alkoxysilane ligand simultaneously tethers the precursor to the support and locks the Cu–M bond during ligand removal, preventing early phase segregation. Cu–Mo and Cu–Fe graft at higher total loadings (0.36–0.68 wt %) and retain a Cu/M in a close to 1:1 ratio, whereas Cu–Ru and Cu–W bind more sparsely (≤0.17 wt %) and evolve into Cu–rich alloys (Cu/M ≈ 1.5–3). Electron microscopy and spectroscopic analyses demonstrated that clusters’ stability is temperature-dependent. Growth of narrowly distributed clusters was detected up to 250 °C. Heating beyond 300 °C accelerates surface diffusion and ultimately triggers phase separation into larger monometallic particles, establishing a practical upper limit for catalytic operation. Smaller clusters’ size and a more homogeneous composition between different clusters may be attained through the application of a rapid annealing procedure, wherein the sample is subjected to a brief (<1 s) exposure duration to an elevated temperature. This approach may provide a potential route toward improved uniformity and will be further explored in a future work.

CuRu and CuW clusters lower the apparent activation energy to ∼45 kJ mol^–1^ in ethylene hydrogenation, which is 10 kJ lower than the activation energy for monometallic Cu nanoparticles. The catalytic properties of Cu–M clusters in ethylene hydrogenation demonstrated composition-dependent reactivity, with Cu–Ru and Cu–W clusters exhibiting lower activation energy than monometallic Cu catalysts. Kinetic studies further revealed alloying influenced reaction mechanisms, particularly for Cu–Ru and Cu–W, where changes in hydrogen and ethylene reaction orders suggested modifications in active site structure and reactivity. The study demonstrates that strong metal–metal bonds embedded in a surface-anchored ligand scaffold are sufficient to nucleate alloy clusters of otherwise immiscible pairs and that precursor-controlled stoichiometry can be translated into catalytic behavior. These findings emphasize the potential of surface-grafted heterobinuclear complexes in achieving control over the composition and size of bimetallic clusters, with potential to yield novel catalytic properties.

## Supplementary Material



## Notes

Additional synthetic and experimental data, including NMR, IR, XPS, and TGA–MS results (PDF)

## References

[1] Liu L. C., Corma A. (2023). Bimetallic
Sites for Catalysis: From Binuclear Metal Sites to Bimetallic
Nanoclusters and Nanoparticles. Chem. Rev..

[2] Alonso D. M., Wettstein S. G., Dumesic J. A. (2012). Bimetallic catalysts for upgrading
of biomass to fuels and chemicals. Chem. Soc.
Rev..

[3] Sankar M., Dimitratos N., Miedziak P. J., Wells P. P., Kiely C. J., Hutchings G. J. (2012). Designing
bimetallic catalysts for a green and sustainable
future. Chem. Soc. Rev..

[4] Paz-Borbon L. O., Johnston R. L., Barcaro G., Fortunelli A. (2008). Structural
motifs, mixing, and segregation effects in 38-atom binary clusters. J. Chem. Phys..

[5] Kumar A., Bui V. Q., Lee J., Wang L. L., Jadhav A. R., Liu X. H., Shao X. D., Liu Y., Yu J. M., Hwang Y., Bui H. T. D., Ajmal S., Kim M. G., Kim S. G., Park G. S., Kawazoe Y., Lee H. (2021). Moving beyond
bimetallic-alloy to single-atom dimer atomic-interface for all-pH
hydrogen evolution. Nat. Commun..

[6] Ferrando R., Jellinek J., Johnston R. L. (2008). Nanoalloys: From
theory to applications
of alloy clusters and nanoparticles. Chem. Rev..

[7] Buchwalter P., Rose J., Braunstein P. (2015). Multimetallic
Catalysis Based on
Heterometallic Complexes and Clusters. Chem.
Rev..

[8] Fan J. X., Du H. X., Zhao Y., Wang Q., Liu Y. N., Li D. Q., Feng J. T. (2020). Recent
Progress on Rational Design
of Bimetallic Pd Based Catalysts and Their Advanced Catalysis. ACS Catal..

[9] Chen M., Gupta G., Ordoñez C., Lamkins A., Ward C., Abolafia C., Zhang B., Roling L. T., Huang W. (2021). Intermetallic
Nanocatalyst for Highly Active Heterogeneous Hydroformylation. J. Am. Chem. Soc..

[10] Dasgupta A., He H. R., Gong R. S., Shang S. L., Zimmerer E. K., Meyer R. J., Liu Z. K., Janik M. J., Rioux R. M. (2022). Atomic
control of active-site ensembles in ordered alloys to enhance hydrogenation
selectivity. Nat. Chem..

[11] He H. R., Canning G. A., Nguyen A., Dasgupta A., Meyer R. J., Rioux R. M., Janik M. J. (2023). Active-site
isolation in intermetallics
enables precise identification of elementary reaction kinetics during
olefin hydrogenation. Nat. Catal..

[12] Lopez-Acevedo O., Kacprzak K. A., Akola J., Hakkinen H. (2010). Quantum size effects
in ambient CO oxidation catalysed by ligand-protected gold clusters. Nat. Chem..

[13] Witham C. A., Huang W. Y., Tsung C. K., Kuhn J. N., Somorjai G. A., Toste F. D. (2010). Converting homogeneous to heterogeneous in electrophilic
catalysis using monodisperse metal nanoparticles. Nat. Chem..

[14] Ye R., Zhukhovitskiy A. V., Deraedt C. V., Toste F. D., Somorjai G. A. (2017). Supported
Dendrimer-Encapsulated Metal Clusters: Toward Heterogenizing Homogeneous
Catalysts. Acc. Chem. Res..

[15] Yang C., Ko B. H., Hwang S., Liu Z., Yao Y., Luc W., Cui M., Malkani A. S., Li T., Wang X., Dai J., Xu B. (2020). Overcoming
Immiscibility Toward Bimetallic
Catalyst Library. Sci. Adv..

[16] Liu S., Dun C., Jiang Q., Xuan Z., Yang F., Guo J., Urban J. J., Swihart M. T. (2024). Challenging Thermodynamics: Combining
Immiscible Elements in a Single-Phase Nano-Ceramic. Nat. Commun..

[17] Xiao S., Hu W., Luo W., Wu Y., Li X., Deng H. (2006). Size Effect
on Alloying Ability and Phase Stability of Immiscible Bimetallic Nanoparticles. Eur. Phys. J. B.

[18] Aslam U., Linic S. (2016). inetic Trapping of Immiscible Metal
Atoms into Bimetallic Nanoparticles
through Plasmonic Visible Light-Mediated Reduction of a Bimetallic
Oxide Precursor: Case Study of Ag–Pt Nanoparticle Synthesis. Chem. Mater..

[19] Li L., Larsen A. H., Romero N. A., Morozov V. A., Glinsvad C., Abild-Pedersen F., Greeley J., Jacobsen K. W., Norskov J. K. (2013). Investigation
of Catalytic Finite-Size-Effects of Platinum Metal Clusters. J. Phys. Chem. Lett..

[20] Luo Z. X., Castleman A. W., Khanna S. N. (2016). Reactivity of Metal Clusters. Chem. Rev..

[21] Sanchez S. I., Menard L. D., Bram A., Kang J. H., Small M. W., Nuzzo R. G., Frenkel A. I. (2009). The Emergence of
Nonbulk Properties
in Supported Metal Clusters: Negative Thermal Expansion and Atomic
Disorder in Pt Nanoclusters Supported on γ-AlO. J. Am. Chem. Soc..

[22] Gross E., Popov I., Asscher M. (2009). Chemical Reactivity of Pd–Au
Bimetallic Nanoclusters Grown via Amorphous Solid Water as Buffer
Layer. J. Phys. Chem. C.

[23] Nijem S., Dery S., Carmiel M., Horesh G., Garrevoet J., Spiers K., Falkenberg G., Marini C., Gross E. (2018). Bimetallic
Pt–Re Nanoporous Networks: Synthesis, Characterization, and
Catalytic Reactivity. J. Phys. Chem. C.

[24] Carmiel-Kostan M., Nijem S., Dery S., Horesh G., Gross E. (2019). Composition–Reactivity
Correlations in Platinum–Cobalt Nanoporous Network as Catalyst
for Hydrodeoxygenation of 5-Hydroxymethylfurfural. J. Phys. Chem. C.

[25] Ashraf S., Liu Y. Y., Wei H. J., Shen R. F., Zhang H. H., Wu X. L., Mehdi S., Liu T., Li B. J. (2023). Bimetallic
Nanoalloy Catalysts for Green Energy Production: Advances in Synthesis
Routes and Characterization Techniques. Small.

[26] Jiang J., Zhou X. L., Lv H. G., Yu H. Q., Yu Y. (2023). Bimetallic-Based
Electrocatalysts for Oxygen Evolution Reaction. Adv. Funct. Mater..

[27] Mondloch J. E., Bayram E., Finke R. G. (2012). A review
of the kinetics and mechanisms
of formation of supported-nanoparticle heterogeneous catalysts. J. Mol. Catal. A:Chem..

[28] Xia X. H., Wang Y., Ruditskiy A., Xia Y. N. (2013). 25th Anniversary
Article: Galvanic Replacement: A Simple and Versatile Route to Hollow
Nanostructures with Tunable and Well-Controlled Properties. Adv. Mater..

[29] Margossian T., Larmier K., Kim S. M., Krumeich F., Müller C., Copéret C. (2017). Supported Bimetallic NiFe Nanoparticles through Colloid
Synthesis for Improved Dry Reforming Performance. ACS Catal..

[30] Laskar M., Skrabalak S. E. (2016). A balancing act: manipulating reactivity of shape-controlled
metal nanocatalysts through bimetallic architecture. J. Mater. Chem. A.

[31] Wang H. W., Wang C. L., Yan H., Yi H., Lu J. L. (2015). Precisely-controlled
synthesis of Au@Pd core-shell bimetallic catalyst via atomic layer
deposition for selective oxidation of benzyl alcohol. J. Catal..

[32] Ding K. L., Cullen D. A., Zhang L. B., Cao Z., Roy A. D., Ivanov I. N., Cao D. M. (2018). A general synthesis
approach for
supported bimetallic nanoparticles via surface inorganometallic chemistry. Science.

[33] Wong A., Liu Q., Griffin S., Nicholls A., Regalbuto J. R. (2017). Synthesis
of ultrasmall, homogeneously alloyed, bimetallic nanoparticles on
silica supports. Science.

[34] Campos J. (2020). Bimetallic
cooperation across the periodic table. Nat.
Rev. Chem..

[35] Powers I. G., Uyeda C. (2017). Metal-Metal Bonds in
Catalysis. ACS Catal..

[36] Copéret C. (2019). Single-Sites
and Nanoparticles at Tailored Interfaces Prepared via Surface Organometallic
Chemistry from Thermolytic Molecular Precursors. Acc. Chem. Res..

[37] Gates B. C., Flytzani-Stephanopoulos M., Dixon D. A., Katz A. (2017). Atomically
dispersed supported metal catalysts: perspectives and suggestions
for future research. Catal. Sci. Technol..

[38] Kulkarni A., Gates B. C. (2009). Spectroscopic Elucidation
of First Steps of Supported
Bimetallic Cluster Formation. Angew. Chem.,
Int. Ed..

[39] Rochlitz L., Searles K., Alfke J., Zemlyanov D., Safonova O. V., Copéret C. (2020). Silica-supported, narrowly distributed,
subnanometric Pt-Zn particles from single sites with high propane
dehydrogenation performance. Chem. Sci..

[40] Copéret C., Comas-Vives A., Conley M. P., Estes D. P., Fedorov A., Mougel V., Nagae H., Núñez-Zarur F., Zhizhko P. A. (2016). Surface
Organometallic and Coordination Chemistry toward
Single-Site Heterogeneous Catalysts: Strategies, Methods, Structures,
and Activities. Chem. Rev..

[41] Flytzani-Stephanopoulos M., Gates B. C. (2012). Atomically
Dispersed Supported Metal Catalysts. Annu. Rev.
Chem. Biomol..

[42] Binding S. C., Pernik I., Gonçales V. R., Wong C. M., Webster R. F., Cheong S., Tilley R. D., Garcia-Bennett A. E., Gooding J. J., Messerle B. A. (2019). Simultaneous Functionalization
of
Carbon Surfaces with Rhodium and Iridium Organometallic Complexes:
Hybrid Bimetallic Catalysts for Hydroamination. Organometallics.

[43] Nozaki C., Lugmair C. G., Bell A. T., Tilley T. D. (2002). Synthesis,
characterization,
and catalytic performance of single-site iron­(III) centers on the
surface of SBA-15 silica. J. Am. Chem. Soc..

[44] Zhang Y. X., Zhang S. B., Huang H. L., Liu X. L., Li B. B., Lee Y., Wang X. D., Bai Y., Sun M. Z., Wu Y. F., Gong S. Y., Liu X. W., Zhuang Z. B., Tan T., Niu Z. Q. (2023). General Synthesis
of a Diatomic Catalyst Library via
a Macrocyclic Precursor-Mediated Approach. J.
Am. Chem. Soc..

[45] Czerny F., Searles K., Sot P., Teichert J. F., Menezes P. W., Copéret C., Driess M. (2021). Well-Defined, Silica-Supported Homobimetallic
Nickel Hydride Hydrogenation Catalyst. Inorg.
Chem..

[46] Bai L. C., Hsu C. S., Alexander D. T. L., Chen H. M., Hu X. L. (2021). Double-atom
catalysts as a molecular platform for heterogeneous oxygen evolution
electrocatalysis. Nat. Energy.

[47] Guo X. Y., Gu J. X., Lin S. R., Zhang S. L., Chen Z. F., Huang S. P. (2020). Tackling the Activity
and Selectivity Challenges of
Electrocatalysts toward the Nitrogen Reduction Reaction via Atomically
Dispersed Biatom Catalysts. J. Am. Chem. Soc..

[48] Hu Y. F., Li Z. S., Li B. L., Yu C. L. (2022). Recent Progress
of Diatomic Catalysts: General Design Fundamentals and Diversified
Catalytic Applications. Small.

[49] Cammarota R. C., Clouston L. J., Lu C. C. (2017). Leveraging molecular
metal-support
interactions for H and N activation. Coordin.
Chem. Rev..

[50] Leon N. J., Yu H. C., Mazzacano T. J., Mankad N. P. (2019). Mixed phosphine/carbonyl
derivatives of heterobimetallic copper-iron and copper-tungsten catalysts. Polyhedron.

[51] Zhong R., Lindhorst A. C., Groche F. J., Kühn F. E. (2017). Immobilization
of N-Heterocyclic Carbene Compounds: A Synthetic Perspective. Chem. Rev..

[52] Mazzacano T. J., Mankad N. P. (2013). Base Metal Catalysts for Photochemical
C-H Borylation
That Utilize Metal-Metal Cooperativity. J. Am.
Chem. Soc..

[53] King R. B. (1970). Some Applications
of Metal Carbonyl Anions in Synthesis of Unusual Organometallic Compounds. Acc. Chem. Res..

[54] Liu J., Chen J., Zhao J., Zhao Y., Li L., Zhang H. (2003). A modified procedure for the synthesis of 1-arylimidazoles. Synthesis.

[55] Behrens U., Edelmann F. (1984). Eine verbesserte Synthese
der Tricarbonyl­(cyclopentadienyl)­metallat-Anionen
des Chroms, Molybdäns und Wolframs. J.
Organomet. Chem..

[56] Banerjee S., Karunananda M. K., Bagherzadeh S., Jayarathne U., Parmelee S. R., Waldhart G. W., Mankad N. P. (2014). Synthesis
and Characterization
of Heterobimetallic Complexes with Direct Cu–M Bonds (M = Cr,
Mn, Co, Mo, Ru, W) Supported by N-Heterocyclic Carbene Ligands: A
Toolkit for Catalytic Reaction Discovery. Inorg.
Chem..

[57] Ohishi T., Shiotani Y., Yamashita M. (1994). A Convenient
One-Flask Preparation
of Pure Potassium Cyclopentadienyldicarbonylferrate, K­[η^5^-(C_5_H_5_)­Fe­(CO)_2_]. J. Org. Chem..

[58] Kalz K. F., Kindermann N., Xiang S.-Q., Kronz A., Lange A., Meyer F. (2014). Revisiting
the Synthesis and Elucidating the Structure of Potassium
Cyclopentadienyldicarbonylruthenate, K­[CpRu­(CO)_2_]. Organometallics.

[59] Tyrrell E., Whiteman L., Williams N. (2011). The synthesis and characterisation
of immobilised palladium carbene complexes and their application to
heterogeneous catalysis. J. Organomet. Chem..

[60] Kleitz F., Choi S. H., Ryoo R. (2003). Cubic Ia3d
Large Mesoporous Silica:
Synthesis and Replication to Platinum Nanowires, Carbon Nanorods and
Carbon Nanotubes. Chem. Commun..

[61] Zhuravlev L. T. (2000). The Surface
Chemistry of Amorphous Silica. Zhuravlev Model. Colloids Surf., A.

[62] Lefort L., Chabanas M., Maury O., Meunier D., Copéret C., Thivolle-Cazat J., Basset J. M. (2000). Versatility of silica
used as a ligand::
effect of thermal treatments of silica on the nature of silica-supported
alkyl tantalum species. J. Organomet. Chem..

[63] Leon N. J., Yu H. C., Mazzacano T. J., Mankad N. P. (2020). Pursuit of C-H Borylation
Reactions with Non-Precious Heterobimetallic Catalysts: Hypothesis-Driven
Variations on a Design Theme. Synlett.

[64] Goellner J. F., Gates B. C., Vayssilov G. N., Rösch N. (2000). Structure
and Bonding of a Site-Isolated Transition Metal Complex: Rhodium Dicarbonyl
in Highly Dealuminated Zeolite Y. J. Am. Chem.
Soc..

[65] Hoffman A. S., Fang C.-Y., Gates B. C. (2016). Homogeneity
of Surface Sites in Supported
Single-Site Metal Catalysts: Assessment with Band Widths of Metal
Carbonyl Infrared Spectra. J. Phys. Chem. Lett..

[66] Ruddy D. A., Jarupatrakorn J., Rioux R. M., Miller J. T., McMurdo M. J., McBee J. L., Tupper K. A., Tilley T. D. (2008). Site-Isolated Pt-SBA15
Materials from Tris­(tert-butoxy)­siloxy Complexes of Pt­(II) and Pt­(IV). Chem. Mater..

[67] Gioffrè D., Rochlitz L., Payard P.-A., Yakimov A., Copéret C. (2022). Grafting of
Group-10 Organometallic Complexes on Silicas: Differences and Similarities,
Surprises and Rationale. Helv. Chim. Acta.

[68] Nishitoba T., Matsumoto K., Ishizaka Y., Arai N., Takeuchi K., Fukaya N., Fujitani T., Endo A., Yasuda H., Sato K., Choi J.-C. (2022). Controlled Growth of Platinum Nanoparticles
on Amorphous Silica from Grafted Pt–Disilicate Complexes. ACS Omega.

[69] Kawawaki T., Kataoka Y., Hirata M., Akinaga Y., Takahata R., Wakamatsu K., Fujiki Y., Kataoka M., Kikkawa S., Alotabi A. S. (2021). Creation of High-Performance
Heterogeneous
Photocatalysts by Controlling Ligand Desorption and Particle Size
of Gold Nanocluster. Angew. Chem., Int. Ed..

[70] Dery S., Berg I., Kim S., Cossaro A., Verdini A., Floreano L., Toste F. D., Gross E. (2020). Strong Metal–Adsorbate
Interactions Increase the Reactivity and Decrease the Orientational
Order of OH-Functionalized N-Heterocyclic Carbene Monolayers. Langmuir.

[71] Dery S., Bellotti P., Ben-Tzvi T., Freitag M., Shahar T., Cossaro A., Verdini A., Floreano L., Glorius F., Gross E. (2021). Influence of N-Substituents on the
Adsorption Geometry of OH-Functionalized
Chiral N-Heterocyclic Carbenes. Langmuir.

[72] Berg I., Amit E., Hale L., Toste F. D., Gross E. (2022). N-Heterocyclic
Carbene Based Nanolayer for Copper Film Oxidation Mitigation. Angew. Chem., Int. Ed..

[73] Campbell J. S., Emmett P. H. (1967). The Catalytic Hydrogenation of Ethylene on Nickel–Copper
and Nickel–Gold Alloys. J. Catal..

[74] Sinfelt J. H. (1973). Specificity
in Catalytic Hydrogenolysis by Metals. Adv.
Catal..

[75] Messervy D. T., Hayes K. E. (1967). Kinetics of Copper-Catalyzed
Hydrogenation of Ethylene. Can. J. Chem..

[76] Heard C. J., Hu C. Q., Skoglundh M., Creaser D., Grönbeck H. (2016). Kinetic Regimes
in Ethylene Hydrogenation over Transition-Metal Surfaces. ACS Catal..

[77] Pirard S. L., Heinrichs B., Heyen G., Pirard J. P. (2008). Optimization
of
experimental procedure and statistical data treatment for kinetics
of ethylene hydrogenation on a copper-magnesia catalyst. Chem. Eng. J..

[78] Moreno-Castilla C., Alvarez-Merino M. A., Carrasco-Marín F., Fierro J. L. G. (2001). Tungsten
and tungsten carbide supported on activated carbon: Surface structures
and performance for ethylene hydrogenation. Langmuir.

[79] Wang L. P., Tysoe W. T. (1991). An Investigation
of Ethylene Hydrogenation Catalyzed
by Metallic Molybdenum Using an Isolatable High-Pressure Reactor -
Identification of the Reaction Site and the Role of Carbonaceous Deposits. J. Catal..

[80] Stacchiola D., Calaza F., Zheng T., Tysoe W. I. (2005). Hydrocarbon
conversion
on palladium catalysts. J. Mol. Catal. A:Chem..

[81] Sprock M., Pruski M., Gerstein B. C., King T. S. (1990). The Effect of Copper
on the Reaction of Ethylene on Silica-Supported Ru-Cu Catalysts as
Studied by C-13 Nmr. Catal. Lett..

