# Supplementary material for: Heterobinuclear Molecular Precursors Direct the Formation of Supported Subnanometer Cu–M Clusters with Tunable Catalytic Behavior
Source: ACS Appl Mater Interfaces. 2025 Sep 24;17(40):56064–76. doi: 10.1021/acsami.5c11995 (PMC12516691; doi:10.1021/acsami.5c11995)
Supplement: Supplementary file 1 [file am5c11995_si_001.pdf]

Supporting information

Heterobinuclear Molecular Precursors Direct the Formation of Supported Sub-nanometer Cu–M Clusters with Tunable Catalytic Behavior

Mazal Kostan-Carmiel[1,2], Hadar Shema[1,2], Hsien-Cheng Yu[3], Griffin A. Canning[4], Dina Shpasser[5], Akshay Soni[5], Sergei Remennik[2], Neal Mankad[3], Robert M. Rioux[4,6], Oz Gazit[5], and Elad Gross[1,2*]

1 Institute of Chemistry, The Hebrew University, Jerusalem 91904, Israel

2 The Center for Nanoscience and Nanotechnology, The Hebrew University, Jerusalem 91904, Israel

3 Department of Chemistry, University of Illinois at Chicago, 845 W. Taylor St., Chicago, Illinois 60607, USA

4 Department of Chemical Engineering, The Pennsylvania State University, University Park, PA, 16802, USA

5 Wolfson Faculty of Chemical Engineering, Technion-Israel Institute of Technology (IIT), Haifa 32000, Israel

6 Department of Chemistry, The Pennsylvania State University, University Park, PA 16802, USA

* Corresponding author email address: elad.gross@mail.huji.ac.il

Table of Contents

1. Supplementary tables…………………………………………………...…….3-6
2. NMR, IR and XPS characterization of the bimetallic complex……........…7-13
3. Image and EDS elemental analysis of the anchored Cu-M complex.……14-40
   3.1. Cu-M (Ru, Fe, W and Mo) clusters prepared at 250 °C…………………14-33
   3.2. Cu-Ru clusters prepared at 200, 300 and 500 °C…………………….......21-29
   3.3. Cu-M clusters structural changes……………………………………..…..30-31
   3.4. In situ cluster formation analysis…………………………………………32-33
4. Ligands desorption and cluster formation analysis……………….…..…34-38
   4.1. TGA-MS measurements...…...34-36
   4.2. Temperature dependent XPS measurements ……..……………………..37
   4.3. In situ CO desorption measurements using DRIFT ………………....…38
5. Catalytic measurements ……………………………………………………39-43
6. References……………………………………………………………….....44

1. Supplementary Tables

Table S1: Surface density of grafted complexes.

**Cu-M sample**	**BET Surface Area ($m^2 \cdot g^{-1}$)**	**Surface density (µmol m^{-2})**	**Molecular Surface density (molecules nm^{-2})**
**CuFe/KIT-6**	600	0.012	0.006
**CuMo/KIT-6**	534	0.020	0.011
**CuRu/KIT-6**	547	0.002	0.001
**CuW/KIT-6**	524	0.004	0.003

Table S2. Atomic percentage of the metals in the grafted CuM and calcined samples.

**Sample**	**Grafted**		**Calcined and reduced**	
	**Cu %**	**M %**	**Cu %**	**M %**
**CuRu**	0.07	0.03	0.08	0.03
**CuFe**	0.79	0.20	0.11	0.30
**CuMo**	1.07	1.01	0.13	0.23
**CuW**	0.09	0.03	0.06	0.04

Table S3. EDS summary of single particle analysis shown in Figures S10-S25

Cu-M Sample	Cu at. %	M at. %	Cu/M at. ratio
**CuRu/KIT-6 @200 °C**	72.9	27.1	2.7 ± 0.3
**CuRu/KIT-6 @250 °C**	57.9	42.1	1.4 ± 0.3
**CuRu/KIT-6 @300 °C**	66.5	33.5	2.0 ± 0.4
**CuRu/KIT-6 @500 °C**	61.2	38.8	1.6 ± 0.4
**CuW/KIT-6 @250 °C**	66.5	33.5	1.9 ± 0.3
**CuMo/KIT-6 @250 °C**	38.2	61.8	0.6 ± 0.2
**CuFe/KIT-6 @250 °C**	48.4	51.6	0.9 ± 0.4

Table S4. Reaction orders in hydrogen and ethylene of monometallic catalyst materials as reported in the literature.

Metal	H_2 order	Ethylene Order
**Cu**[1]	1	0
**Ru**[2]	0.2	0.4
**Mo**[3]	1.1	0
**Fe**[4]	0.6	>0, small
**W**	--[a]	--[a]

[a] No values for ethylene and hydrogen reaction orders were found in the literature for W-based catalysts.

Supplementary Figures

2. NMR, IR and XPS characterization of the bimetallic complex

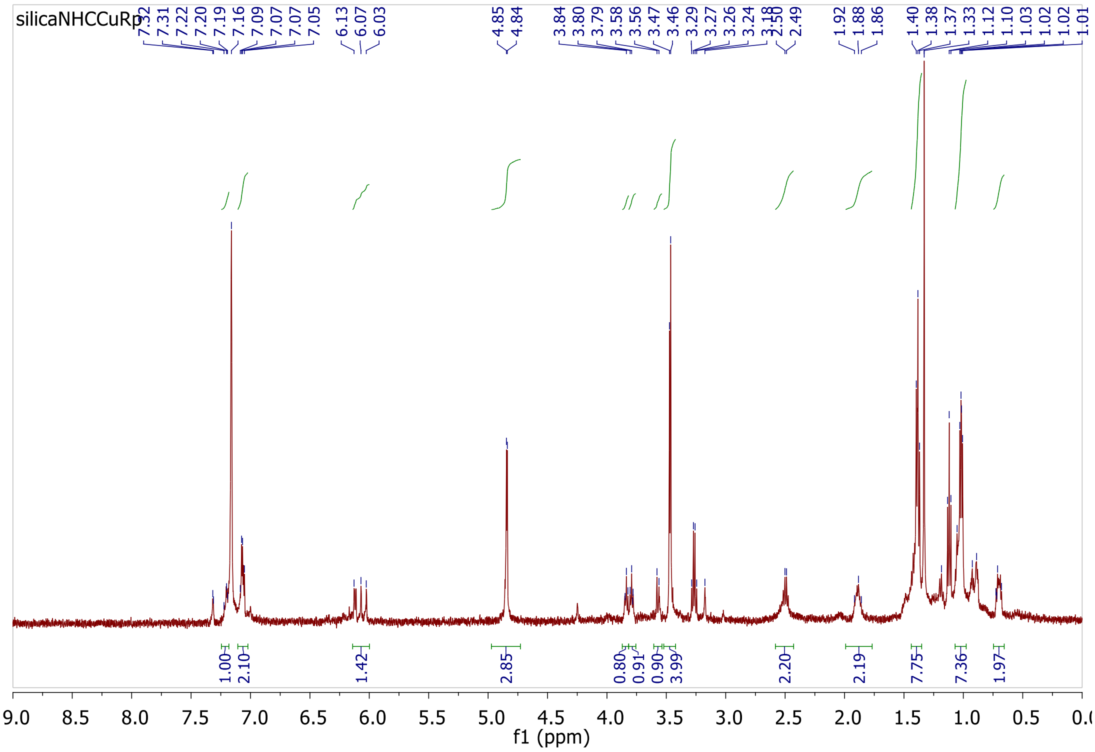

Figure S1. ^{1}H NMR spectrum of Cu-Ru heterobinuclear complex.

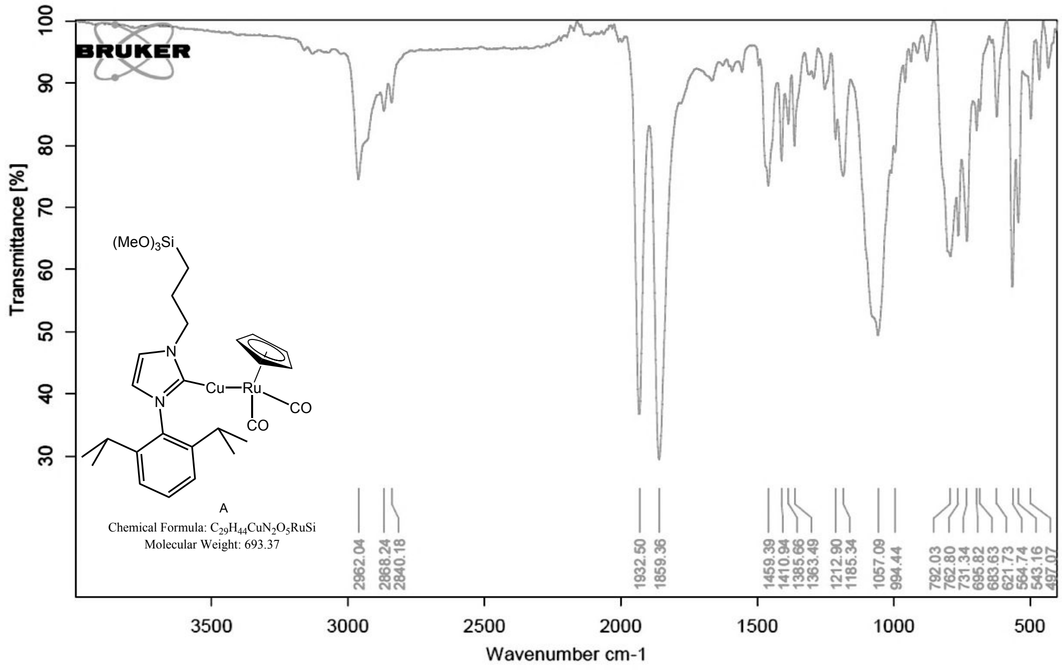

Figure S2. IR spectrum for Cu-Ru heterobinuclear complex.

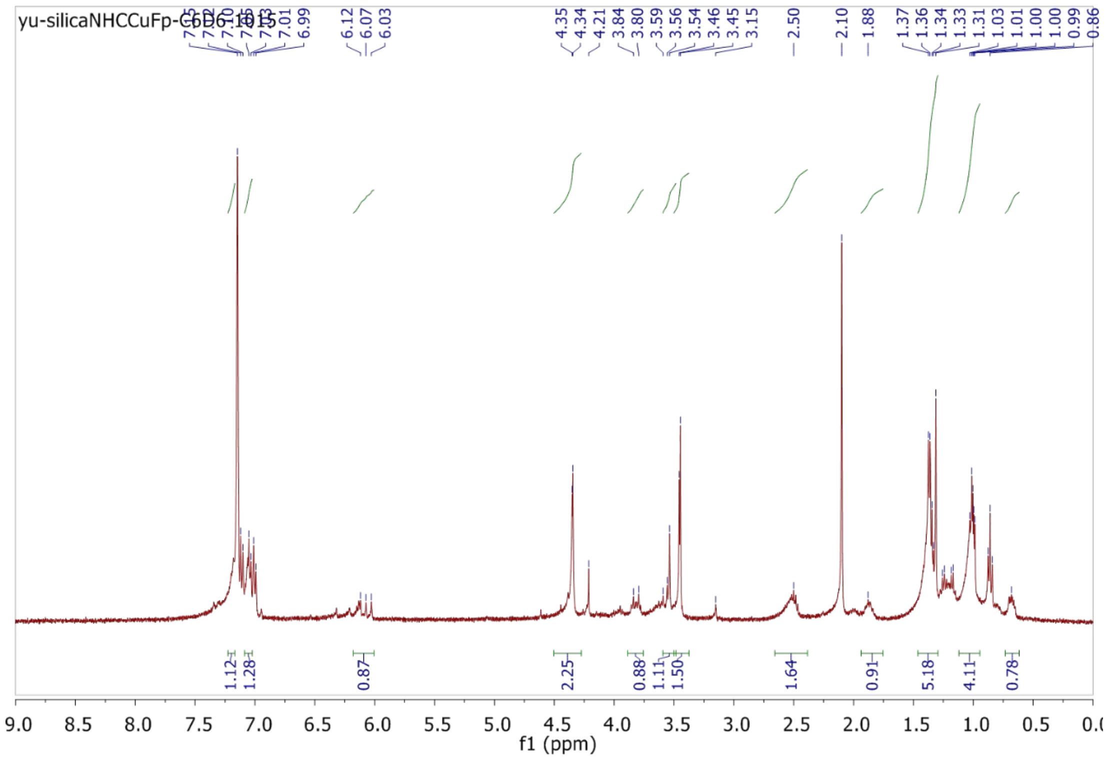

Figure S3. ^{1}H NMR spectrum for Cu-Fe heterobinuclear complex.

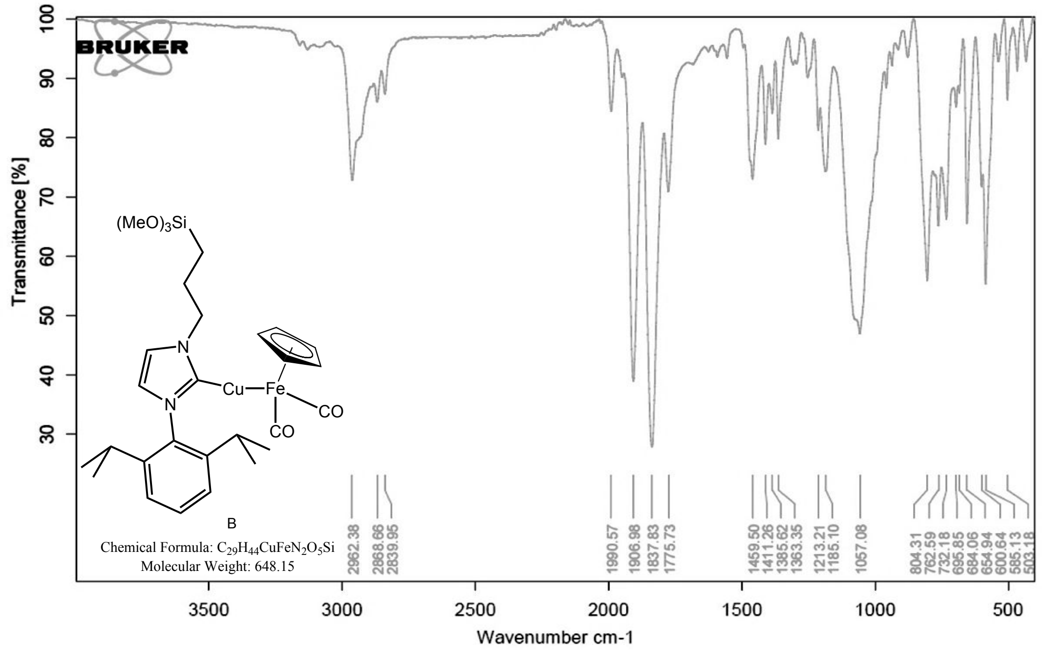

Figure S4. IR spectrum for Cu-Fe heterobinuclear complex.

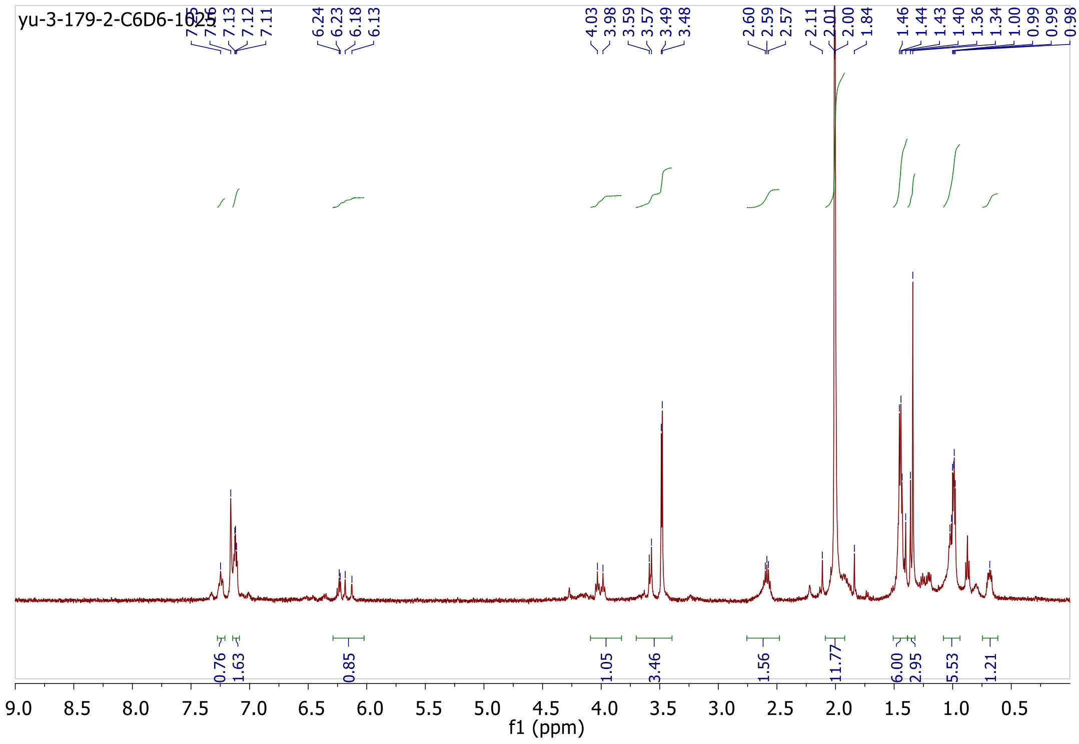

Figure S5. ^{1}H NMR spectrum for Cu-W heterobinuclear complex.

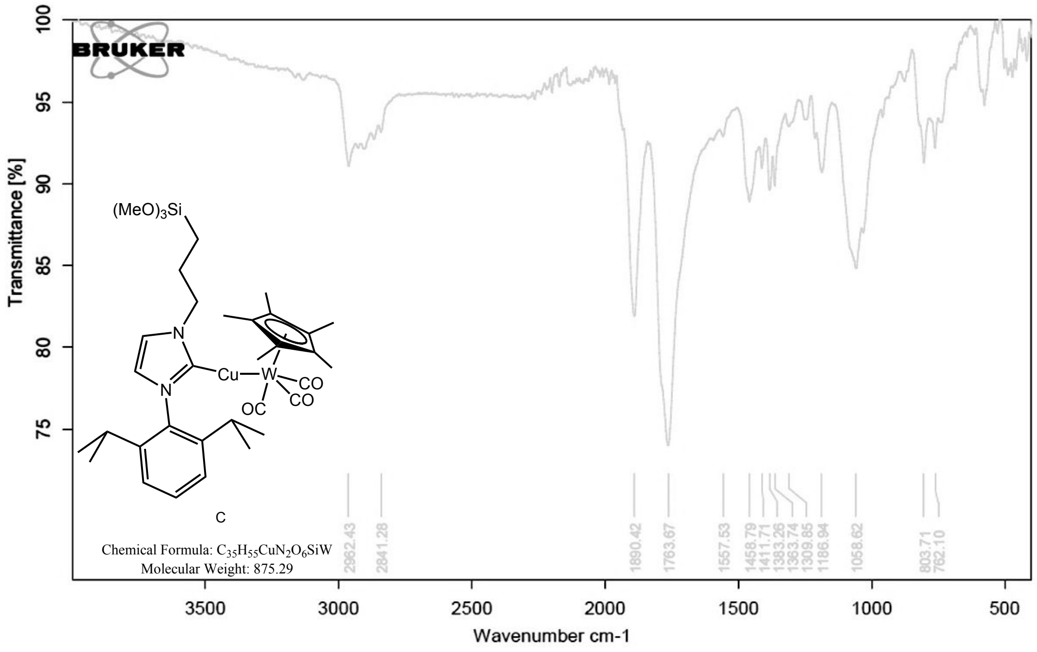

Figure S6. IR spectrum for Cu-W heterobinuclear complex.

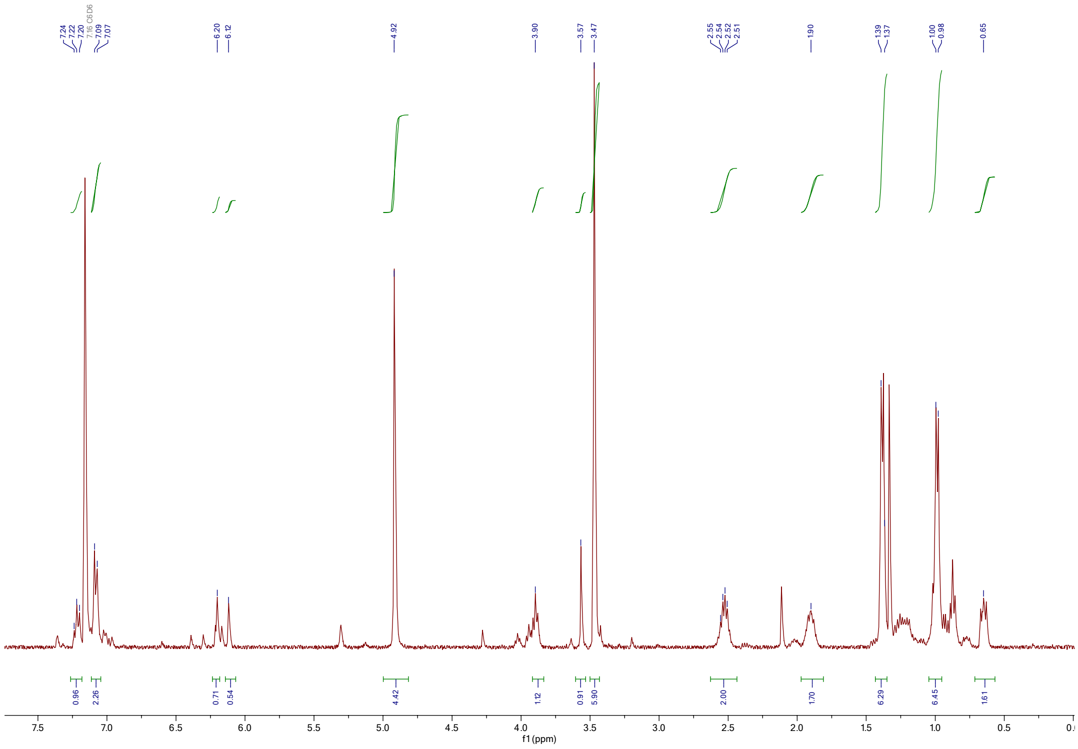

Figure S7. ^{1}H NMR spectrum for Cu-Mo heterobinuclear complex.

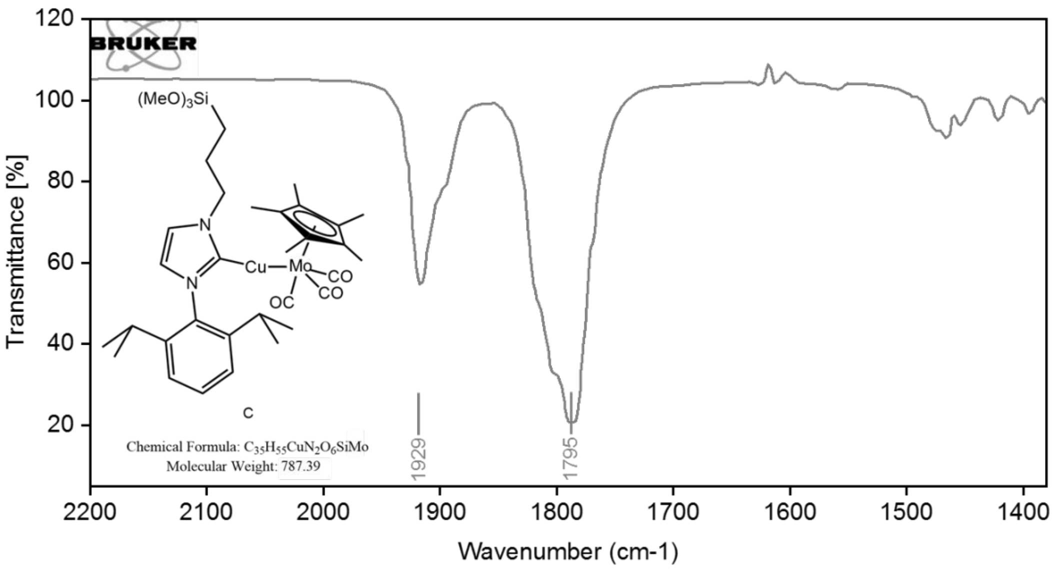

Figure S8. IR spectrum for Cu-Mo heterobinuclear complex.

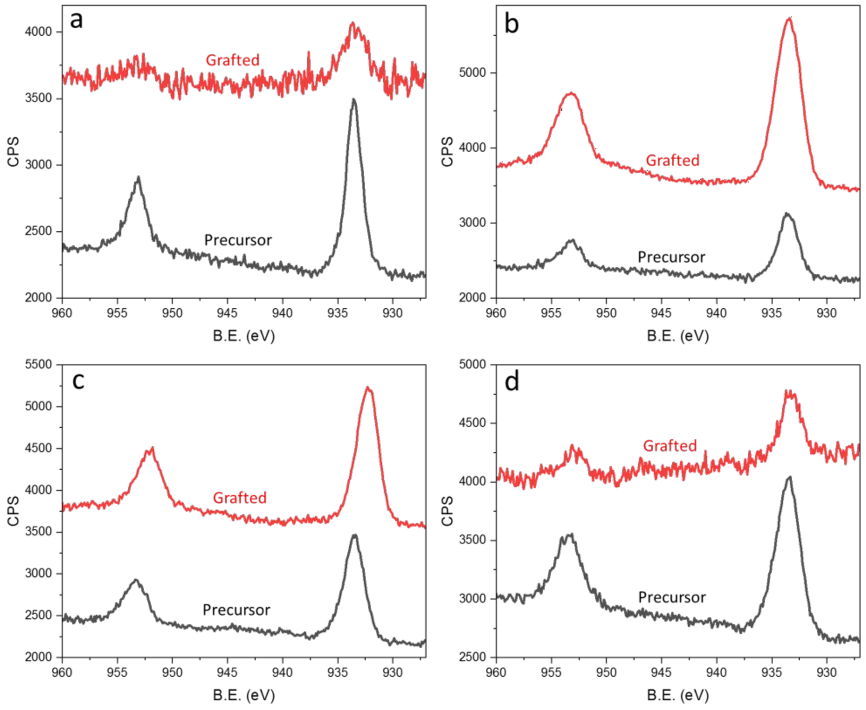

Figure S9. Cu2p XPS signals of the precursor and grafted complex. (**a**) CuW, (**b**) CuFe, (**c**) CuMo and (**d**) CuRu.

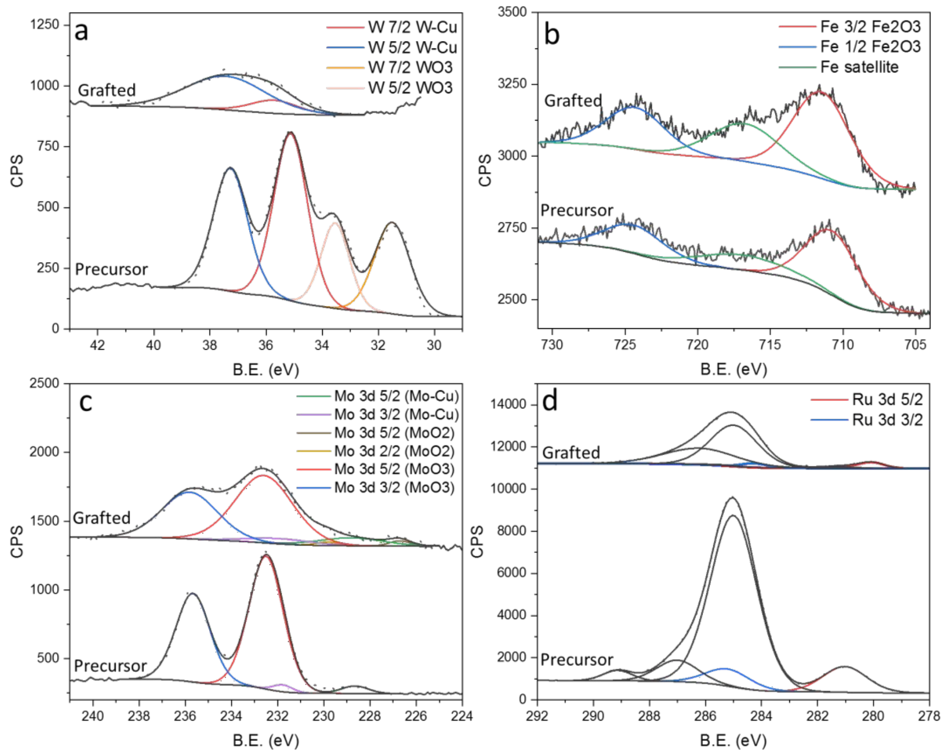

Figure S10. XPS signals of M (W4f, Fe2p, Mo3d and Ru3d) in the Cu-M precursor and the grafted complex. (**a**) CuW, (**b**) CuFe, (**c**) CuMo and (**d**) CuRu.

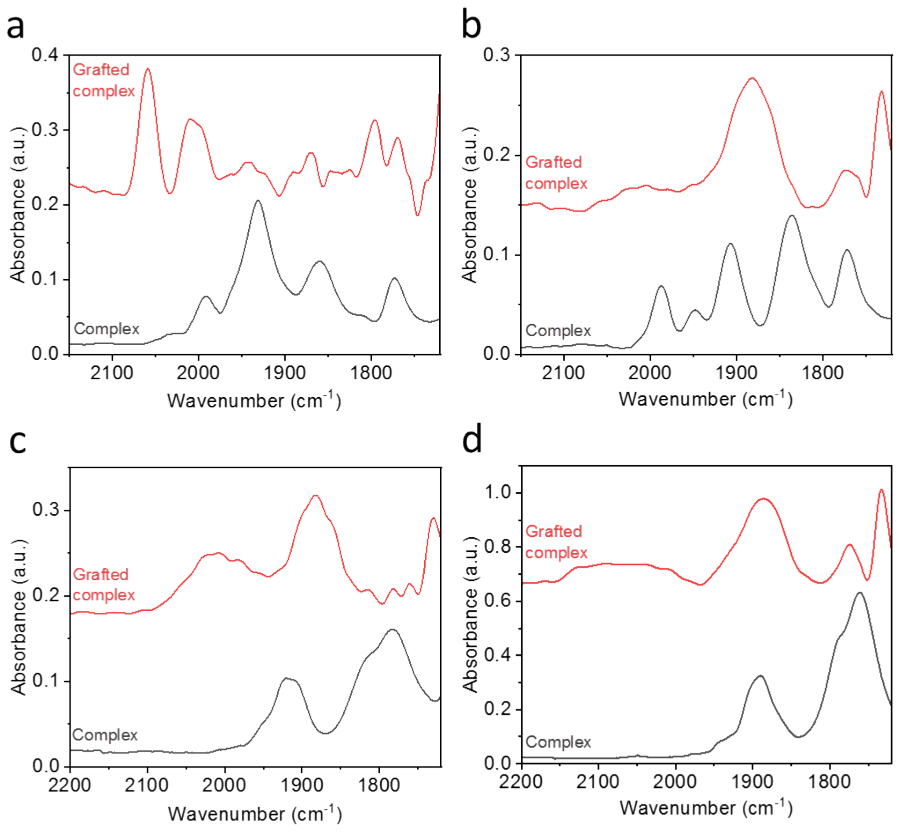

Figure S11. ATR (black-colored) and DRIFT (red-colored) spectra of (NHC)Cu-[M_{CO}] precursors and grafted L-Cu-M, respectively, while M = Ru (**a**), Fe (**b**), Mo (**c**), and W (**d**).

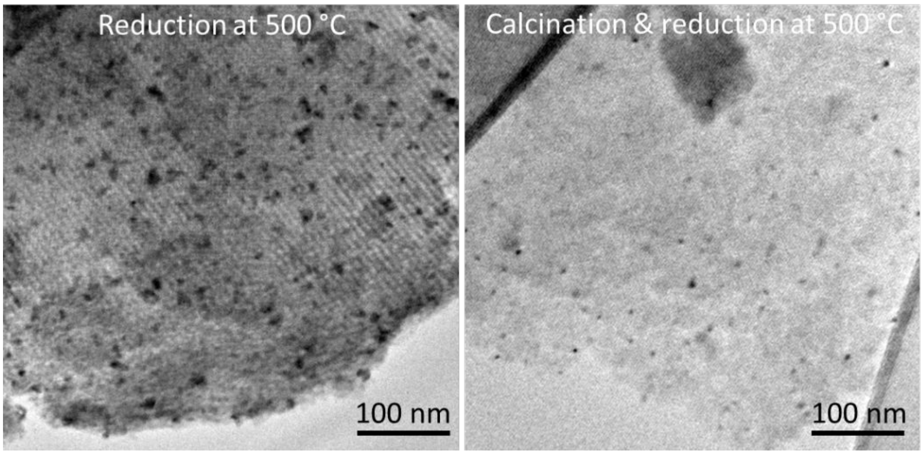

Figure S12: TEM images of supported Cu–Ru clusters that were prepared by reduction at 500 °C (left) and by calcination followed by reduction at 500 °C (right).

3. Image and EDS elemental analysis of the Cu-M complex

3.1. Cu-M (Ru, Fe, W, and Mo) clusters prepared at 250 °C

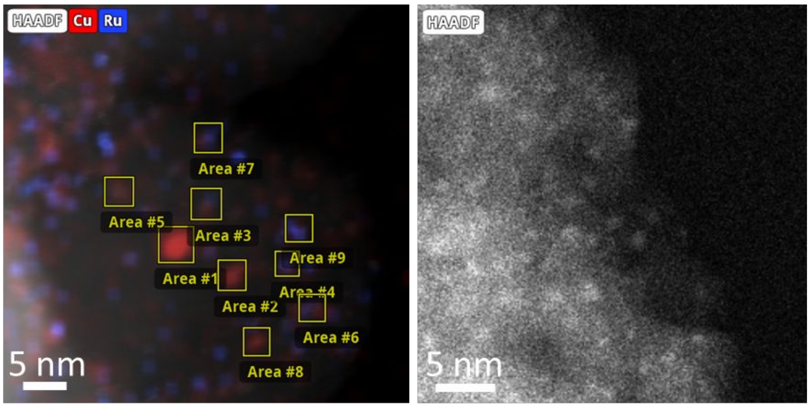

Figure S13. Single particle EDS elemental mapping of Cu (red) and Ru (blue) following calcination and reduction at 250 °C.

Single-NP EDS analysis of Cu-Ru clusters from Figure S10.

Site	Cu (atomic %)	Ru (atomic %)
Particle 1	99.4	0.6
Particle 2	95.5	4.5
Particle 3	53.4	46.6
Particle 4	45.1	54.9
Particle 5	96.5	3.5
Particle 6	45.6	54.4
Particle 7	34.9	65.1
Particle 8	91.7	8.3
Particle 9	30.8	69.2
Particles average	65.8	34.2

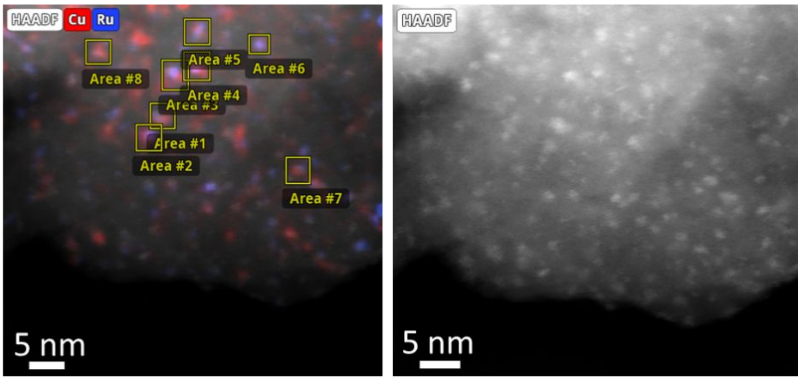

Figure S14. Single particle EDS elemental mapping of Cu (red) and Ru (blue) following calcination and reduction at 250 °C.

Single-NP EDS analysis of Cu-Ru clusters from Figure S11.

Site	Cu (atomic %)	Ru (atomic %)
Particle 1	57.2	42.8
Particle 2	52.2	47.8
Particle 3	53.6	46.4
Particle 4	62.8	37.2
Particle 5	47.5	52.5
Particle 6	40.4	59.6
Particle 7	56.1	43.9
Particle 8	69.7	30.3
Particles average	54.5	45.5

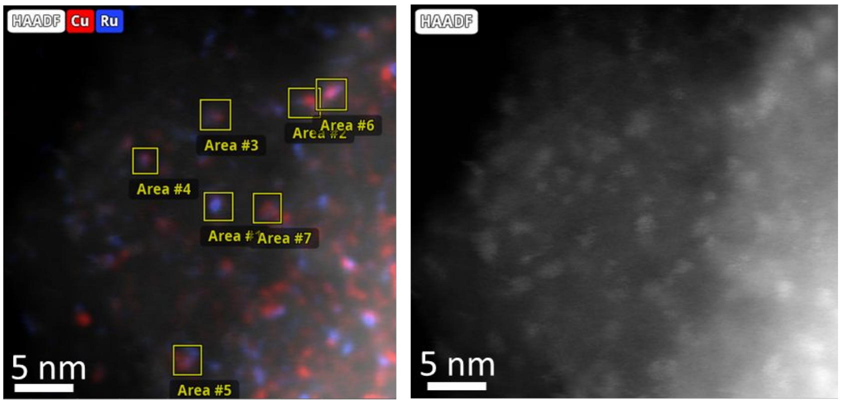

Figure S15. Single particle EDS elemental mapping of Cu (red) and Ru (blue) following calcination and reduction at 250 °C.

Single-NP EDS analysis of Cu-Ru clusters from Figure S12.

Site	Cu (atomic %)	Ru (atomic %)
Particle 1	35.9	64.1
Particle 2	54.1	45.9
Particle 3	50	50
Particle 4	53.3	46.7
Particle 5	50	50
Particle 6	50	50
Particle 7	64.3	35.7
Particles average	51	49

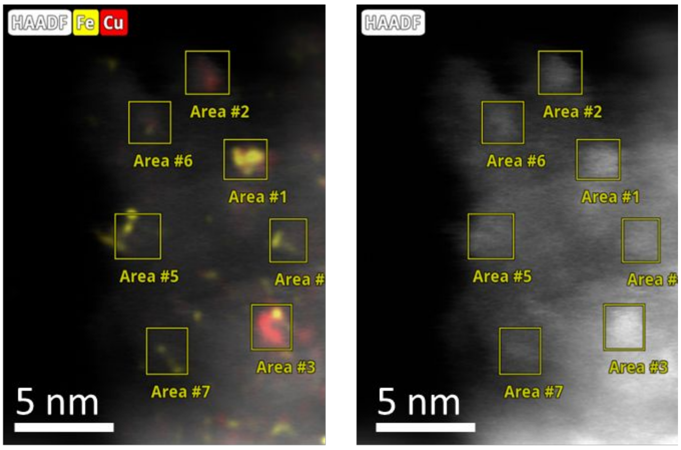

Figure S16. Single particle EDS elemental mapping of Cu (red) and Fe (yellow) following calcination and reduction at 250 °C.

Single-NP EDS analysis of Cu-Fe clusters reduced at 250°C from Figure S13.

Site	Cu (atomic %)	Fe (atomic %)
Particle 1	65.5	34.5
Particle 2	88.2	11.8
Particle 3	88.5	11.5
Particle 4	53.6	46.4
Particle 5	42.8	57.2
Particle 6	75.8	24.2
Particle 7	36.2	63.8
Particles average	64.4	35.6

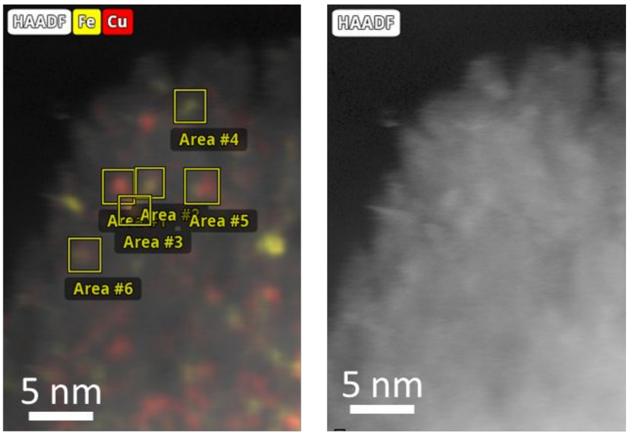

Figure S17. Single particle EDS elemental mapping of Cu (red) and Fe (yellow) following calcination and reduction at 250 °C.

Single-NP EDS analysis of Cu-Fe clusters from Figure S14.

Site	Cu (atomic %)	Fe (atomic %)
Particle 1	47.5	52.5
Particle 2	27.2	72.8
Particle 3	29.7	70.3
Particle 4	8.5	91.5
Particle 5	35.9	64.1
Particle 6	29.7	70.3
Particles average	29.8	70.2

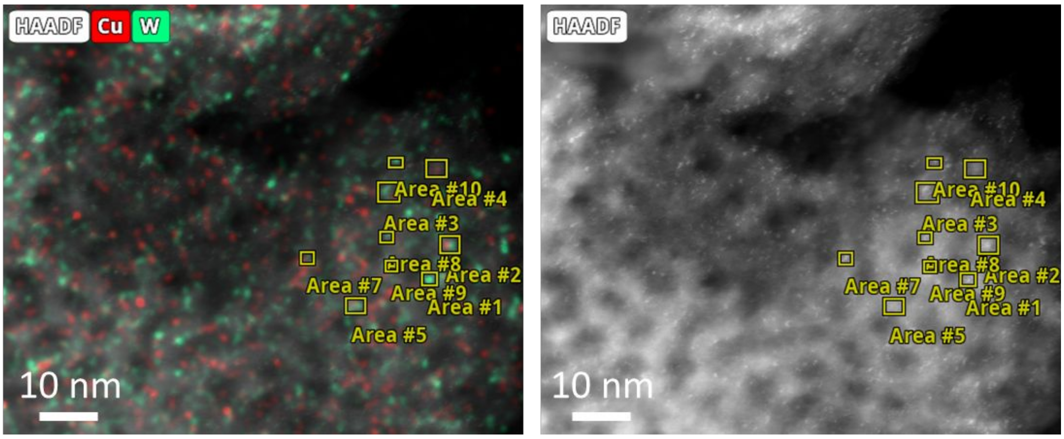

Figure S18. Single particle EDS elemental mapping of Cu (red) and W (green) following calcination and reduction at 250 °C.

Single-NP EDS analysis of Cu-W clusters from Figure S15.

Site	Cu (atomic %)	W (atomic %)
Particle 1	97.8	2.2
Particle 2	48.7	51.3
Particle 3	71.8	28.2
Particle 4	19.9	80.1
Particle 5	57.1	42.9
Particle 6	45.3	54.7
Particle 7	86.7	13.3
Particle 8	76.6	23.4
Particle 9	94.7	5.3
Particles average	66.5	33.5

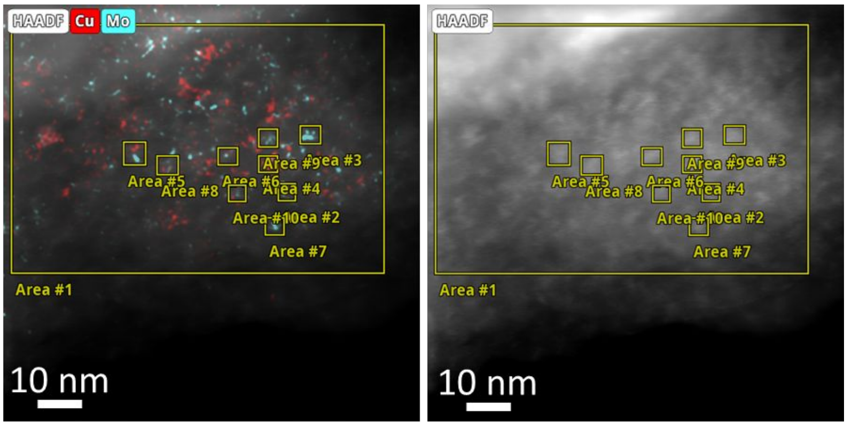

Figure S19. Single particle EDS elemental mapping of Cu (red) and Mo (turquois) following calcination and reduction at 250 °C.

Single-NP EDS analysis of Cu-Mo clusters from Figure S16.

Site	Cu (atomic %)	Mo (atomic %)
Particle 2	34.7	65.3
Particle 3	32.1	67.9
Particle 4	46.9	53.1
Particle 5	43.4	56.6
Particle 6	43.1	56.9
Particle 7	24.5	75.5
Particle 8	42.7	57.3
Particle 9	34.3	65.7
Particle 10	41.7	58.3
Particles average	38.2	61.8

3.2. CuRu clusters prepared at 200, 300 and 500 °C

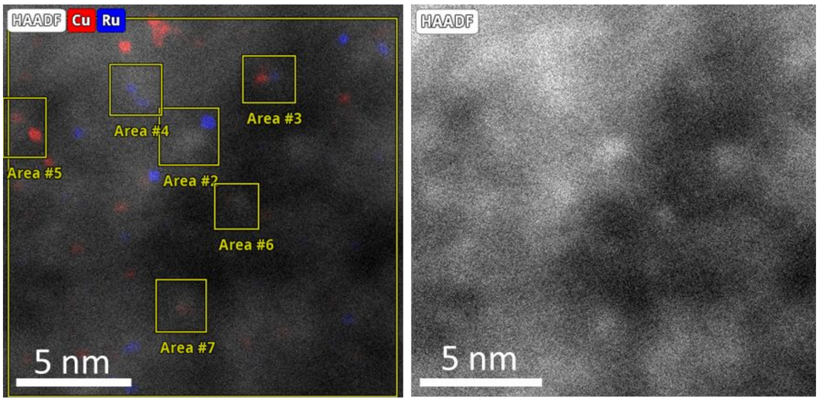

Figure S20. Single particle EDS elemental mapping of Cu (red) and Ru (blue) following calcination and reduction at 200 °C.

Single-NP EDS analysis of Cu-Ru clusters from Figure S17.

Site	Cu (atomic %)	Ru (atomic %)
Particle 2	41	59
Particle 3	57	43
Particle 4	53	47
Particle 5	86	14
Particle 6	97	3
Particle 7	99	1
Particles average	69	31

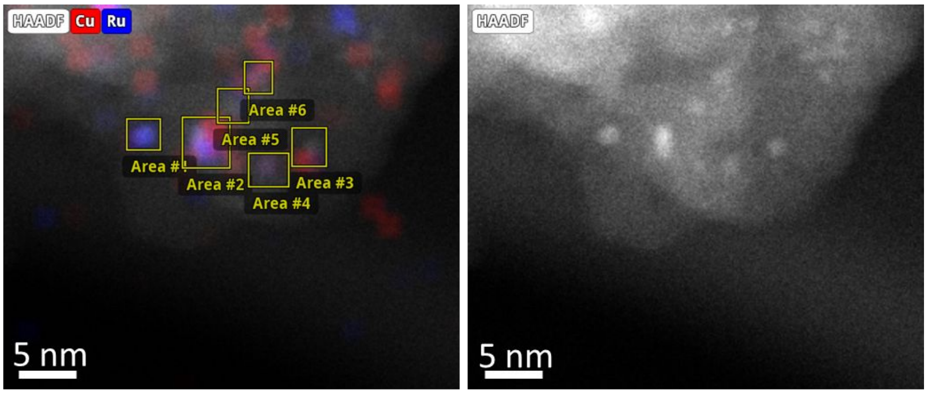

Figure S21. Single particle EDS elemental mapping of Cu (red) and Ru (blue) following calcination and reduction at 300 °C.

Single-NP EDS analysis of Cu-Ru clusters from Figure S18.

Site	Cu (atomic %)	Ru (atomic %)
Particle 1	7.5	92.5
Particle 2	54	46
Particle 3	99.3	0.7
Particle 4	99.8	0.2
Particle 5	99.7	0.3
Particle 6	92.8	7.2
Particles average	75.5	24.5

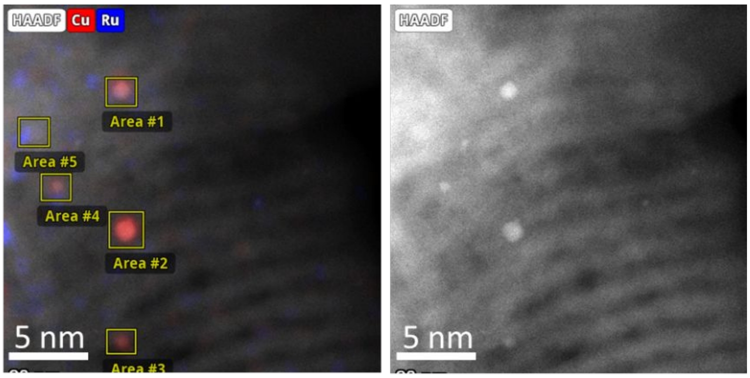

Figure S22. Single particle EDS elemental mapping of Cu (red) and Ru (blue) following calcination and reduction at 300 °C.

Single-NP EDS analysis of Cu-Ru clusters from Figure S19.

Site	Cu (atomic %)	Ru (atomic %)
Particle 1	89.8	10.2
Particle 2	99.1	0.9
Particle 3	94.3	5.7
Particle 4	85.1	14.9
Particle 5	50	50
Particles average	83.6	16.4

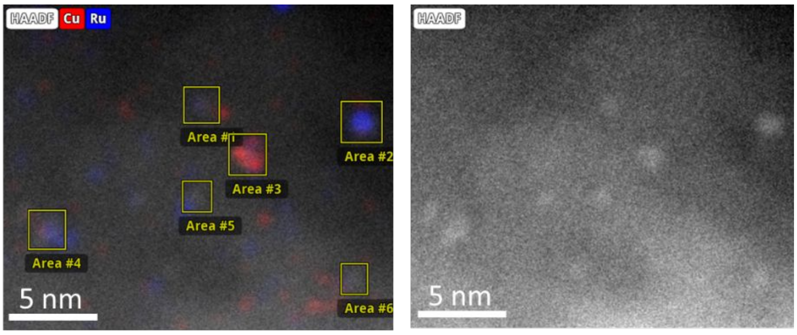

Figure S23. Single particle EDS elemental mapping of Cu (red) and Ru (blue) following calcination and reduction at 300 °C.

Single-NP EDS analysis of Cu-Ru clusters from Figure S20.

Site	Cu (atomic %)	Ru (atomic %)
Particle 1	51.7	48.3
Particle 2	25.4	74.6
Particle 3	72.7	27.3
Particle 4	31.7	68.3
Particle 5	0.5	99.5
Particle 6	54.7	45.3
Particles average	39.5	60.5

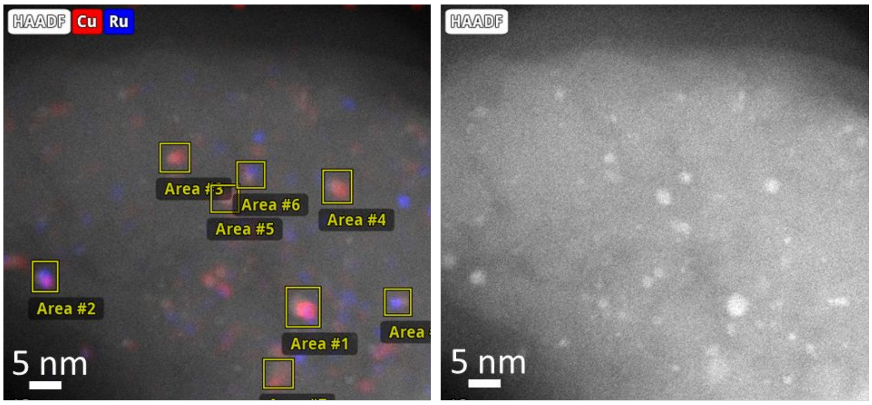

Figure S24. Single particle EDS elemental mapping of Cu (red) and Ru (blue) following calcination and reduction at 300 °C.

Single-NP EDS analysis of Cu-Ru clusters from Figure S21.

Site	Cu (atomic %)	Ru (atomic %)
Particle 1	93.4	6.6
Particle 2	59.5	40.5
Particle 3	91.9	8.1
Particle 4	92.4	7.6
Particle 5	82.4	17.6
Particle 6	62.3	37.7
Particle 7	94.2	5.8
Particle 8	78.8	21.2
Particles average	81.8	18.2

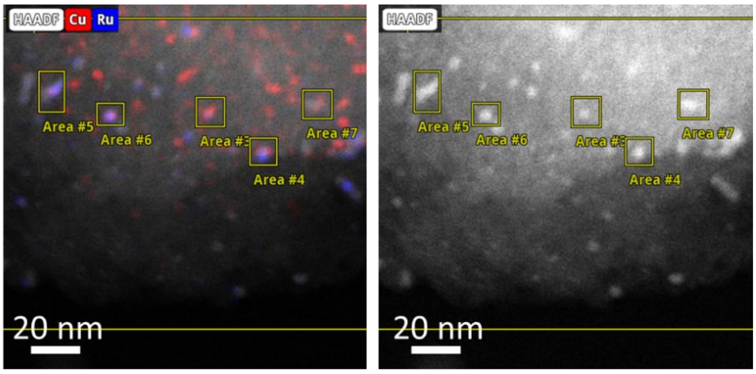

Figure S25. Single particle EDS elemental mapping of Cu (red) and Ru (blue) following calcination and reduction at 500 °C.

Single-NP EDS analysis of Cu-Ru clusters from Figure S22.

Site	Cu (atomic %)	Ru (atomic %)
Particle 3	74.5	25.5
Particle 4	39.4	60.6
Particle 5	28.7	71.3
Particle 6	50.1	49.9
Particle 7	46.2	53.8
Particles average	47.8	52.2

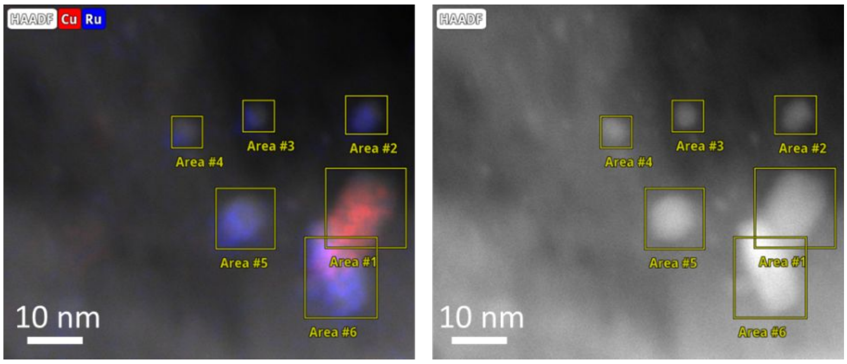

Figure S26. Single particle EDS elemental mapping of Cu (red) and Ru (blue) following calcination and reduction at 500 °C.

Single-NP EDS analysis of Cu-Ru clusters from Figure S23.

Site	Cu (atomic %)	Ru (atomic %)
Particle 1	99.4	0.6
Particle 2	42.4	57.6
Particle 3	22.4	77.6
Particle 4	47.2	52.8
Particle 5	38.2	61.8
Particle 6	70.8	29.2
Particles average	53.4	46.6

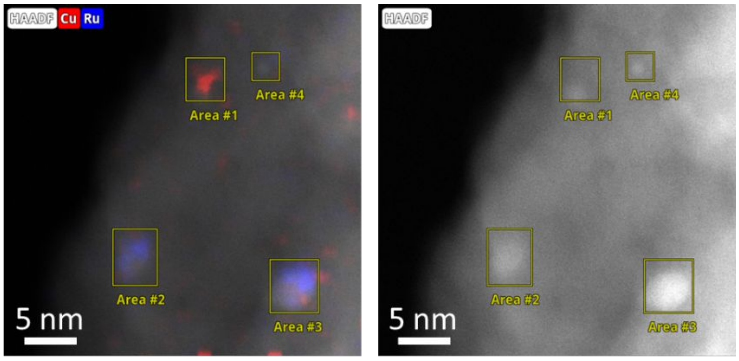

Figure S27. Single particle EDS elemental mapping of Cu (red) and Ru (blue) following calcination and reduction at 500 °C.

Single-NP EDS analysis of Cu-Ru clusters from Figure S24.

Site	Cu (atomic %)	Ru (atomic %)
Particle 1	96.2	3.8
Particle 2	41.7	58.3
Particle 3	28.6	71.4
Particle 4	38.3	61.7
Particles average	51.2	48.8

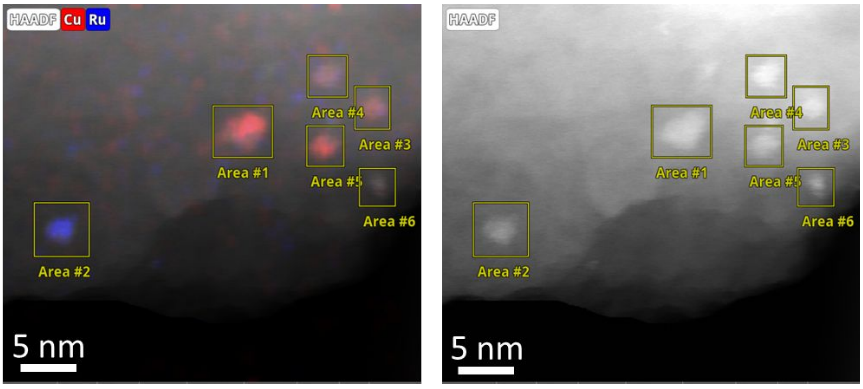

Figure S28. Single particle EDS elemental mapping of Cu (red) and Ru (blue) following calcination and reduction at 500 °C.

Single-NP EDS analysis of Cu-Ru clusters from Figure S25.

Site	Cu (atomic %)	Ru (atomic %)
Particle 1	99.1	0.9
Particle 2	54.6	45.4
Particle 3	97.4	2.6
Particle 4	85.2	14.8
Particle 5	97.2	2.8
Particle 6	87.4	12.6
Particles average	86.8	13.2

3.3. Cu-M clusters structural changes

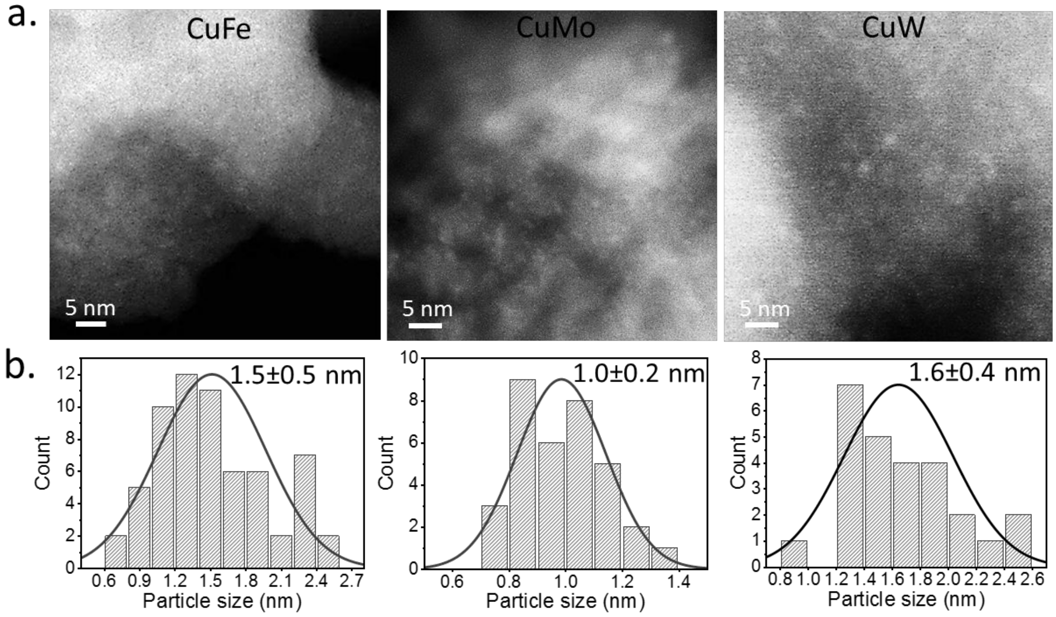

Figure S29. (**a**) HAADF-STEM images of Cu-M clusters where M corresponds to Fe, Mo and W from left to right, respectively. The bimetallic clusters were calcined and reduced at 300 °C. (**b**) The corresponding size distribution analysis of the clusters shown in **a**.

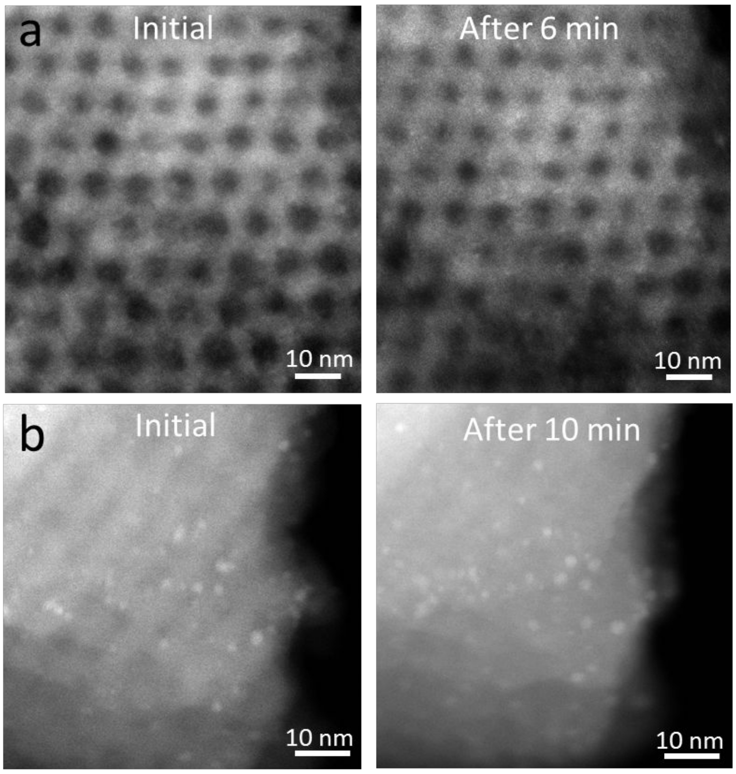

Figure S30. HAADF-STEM images demonstrate the structural changes that are induced after prolonged exposure to the electron beam. CuRu clusters were calcined and reduced at **(a)** 250 °C and **(b)** 300 °C.

3.4. In-situ cluster formation analysis

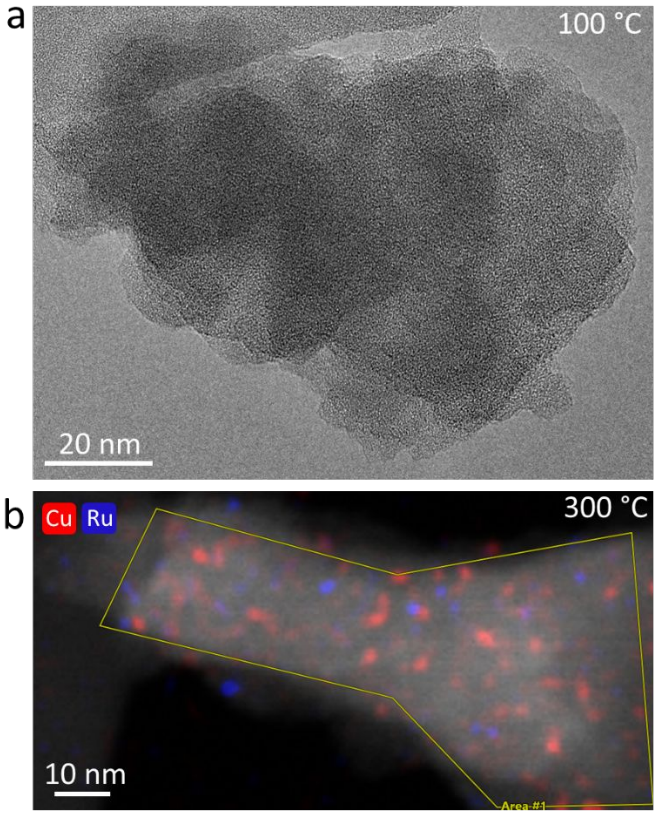

Figure S31. (a) TEM image of Cu-Ru complex after annealing to 100 °C. **(b)** HAADF-STEM mapping of Cu (red) and Ru (blue) after annealing to 300 °C.

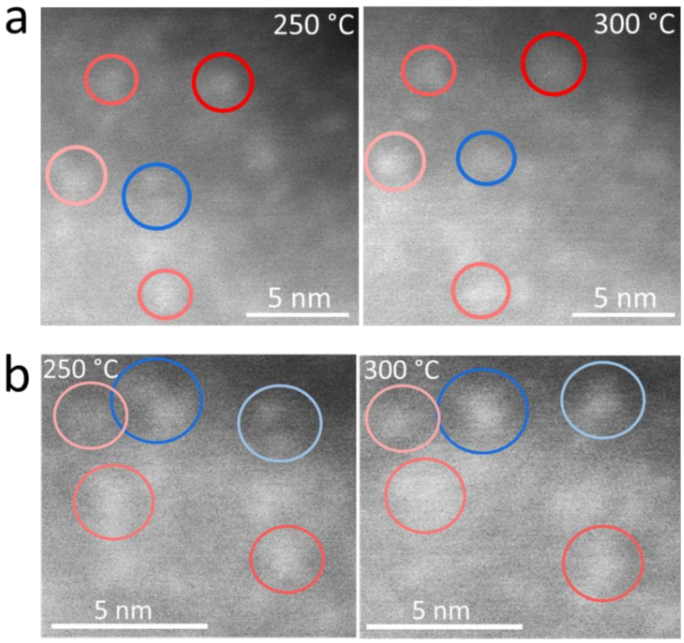

Figure S32. In-situ HAADF-STEM imaging of grafted Cu-Ru complexes on silica under annealing to 250 and 300 °C. Particle growth is demonstrated in two regions, marked as **(a)** and **(b)**. Blue and light blue circles indicate coalescence of two sub-nanometer clusters, and red and light red circles indicate coalescence of smaller species.

4. Ligand desorption and cluster formation analysis

4.1. TGA-MS measurements

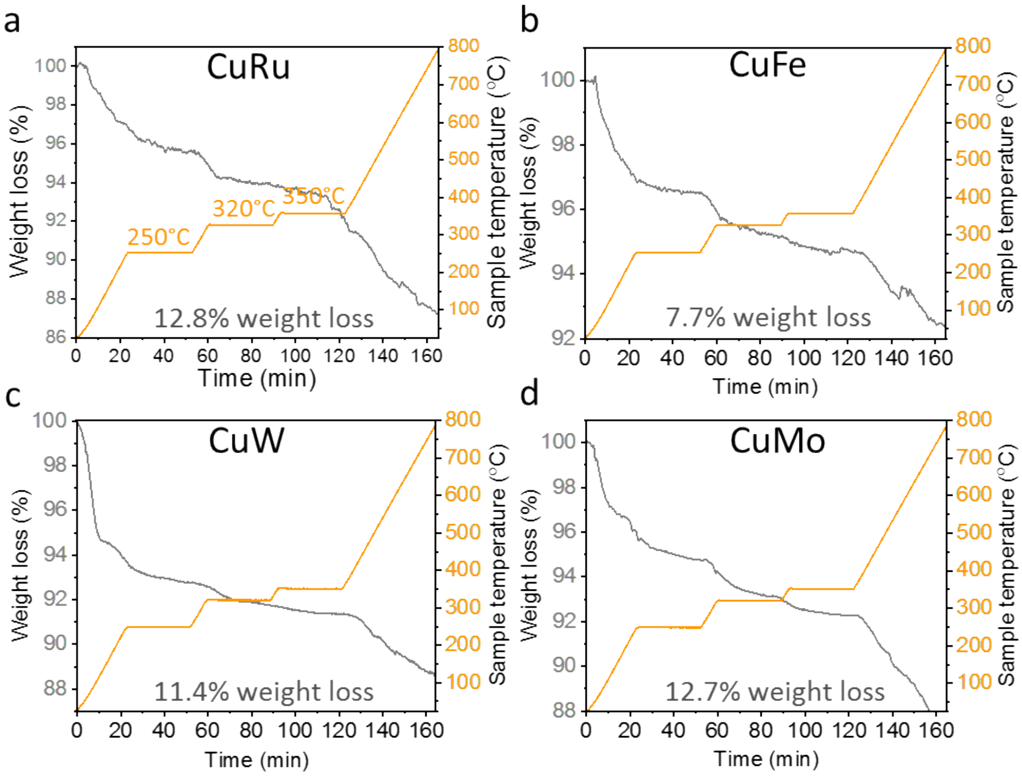

Figure S33. TGA weight loss measurements monitoring the grafted ligand desorption of (**a**) $RuCp(CO)_2$, (**b**) $FeCp(CO)_2$, (**c**) $WCp(CO)_3$ and (**d**) $MoCp(CO)_3$, that were supported on KIT-6.

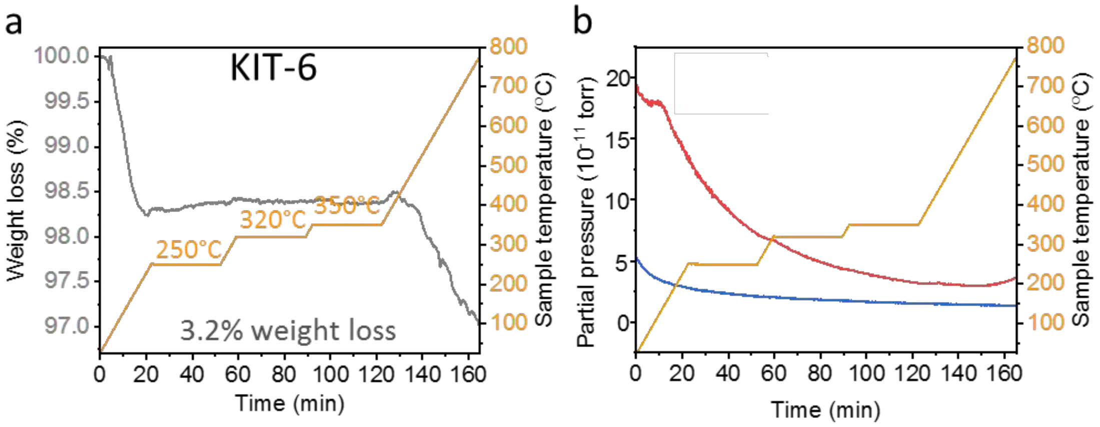

Figure S34. TGA-MS analysis of KIT-6. (**a**) Weight loss and the resulting (**b**) CO_2 (red) and NO (blue) signals as a function of temperature.

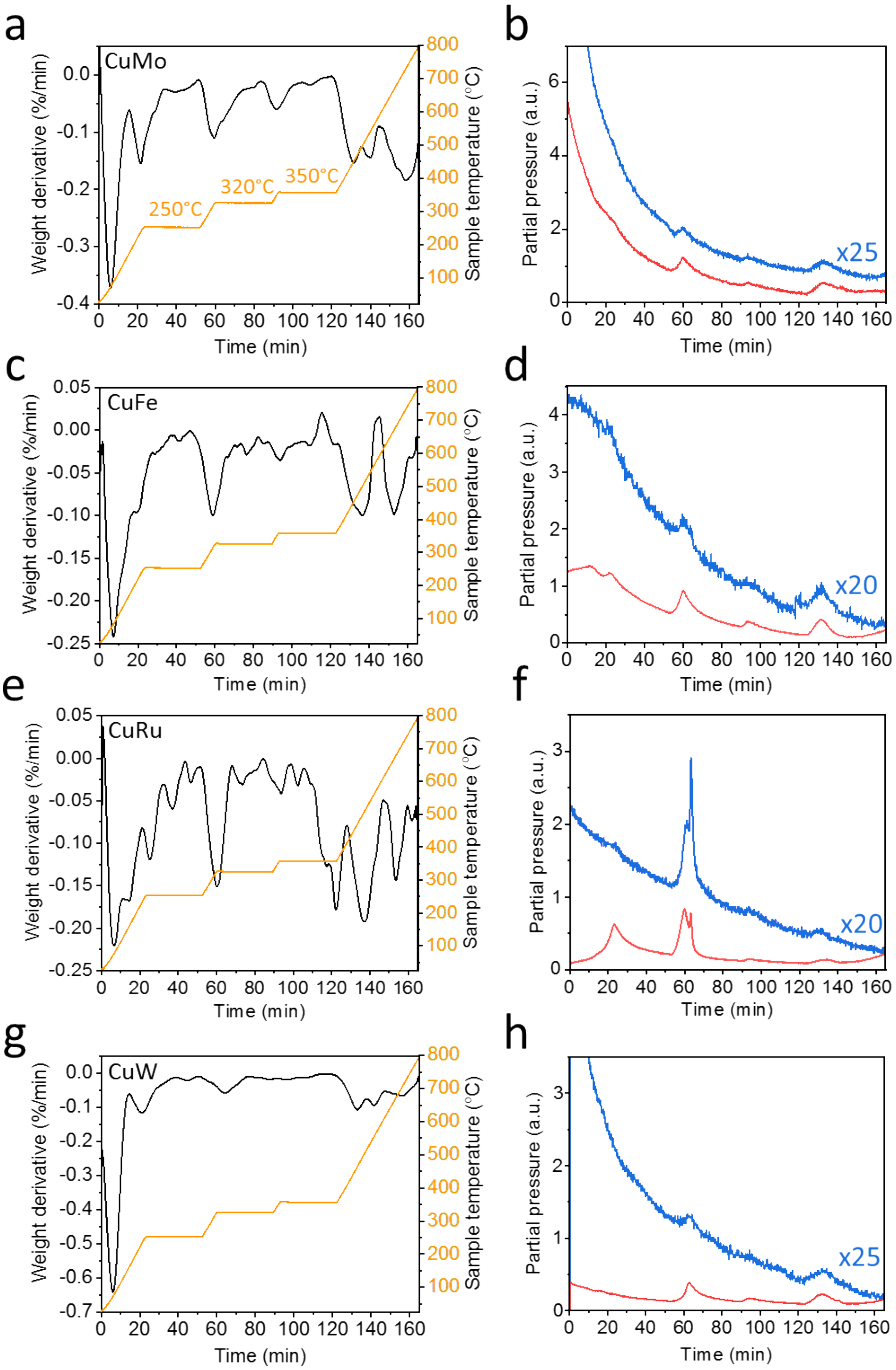

Figure S35. TGA-MS measurements of the grafted complex. TGA-mass loss derivative for CuMo, CuFe, CuRu and CuW (**a, c, e** and **g**, respectively). CO_2 (red-colored) and NO (blue-colored) signals as a function of temperature for CuMo, CuFe, CuRu and CuW (**b, d, f** and **h**, respectively). The NO signal was multiplied by 20 (**d** and **f**) and 25 (**b** and **h**).

4.2. Temperature dependents XPS measurements

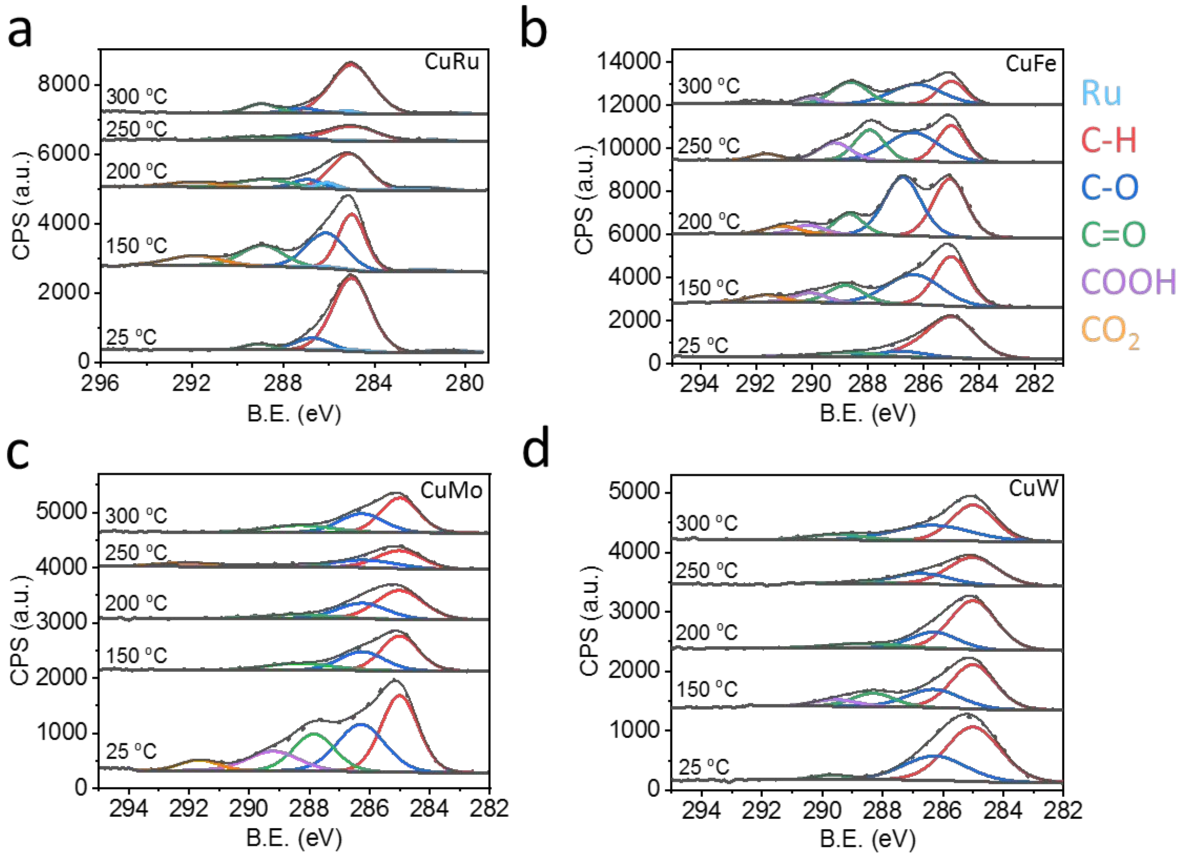

Figure S36. Temperature dependent C1s XPS measurements of grafted (**a**) CuRu, (**b**) CuFe, (**c**) CuMo and (**d**) CuW complexes.

4.3. In situ CO desorption measurements using DRIFT

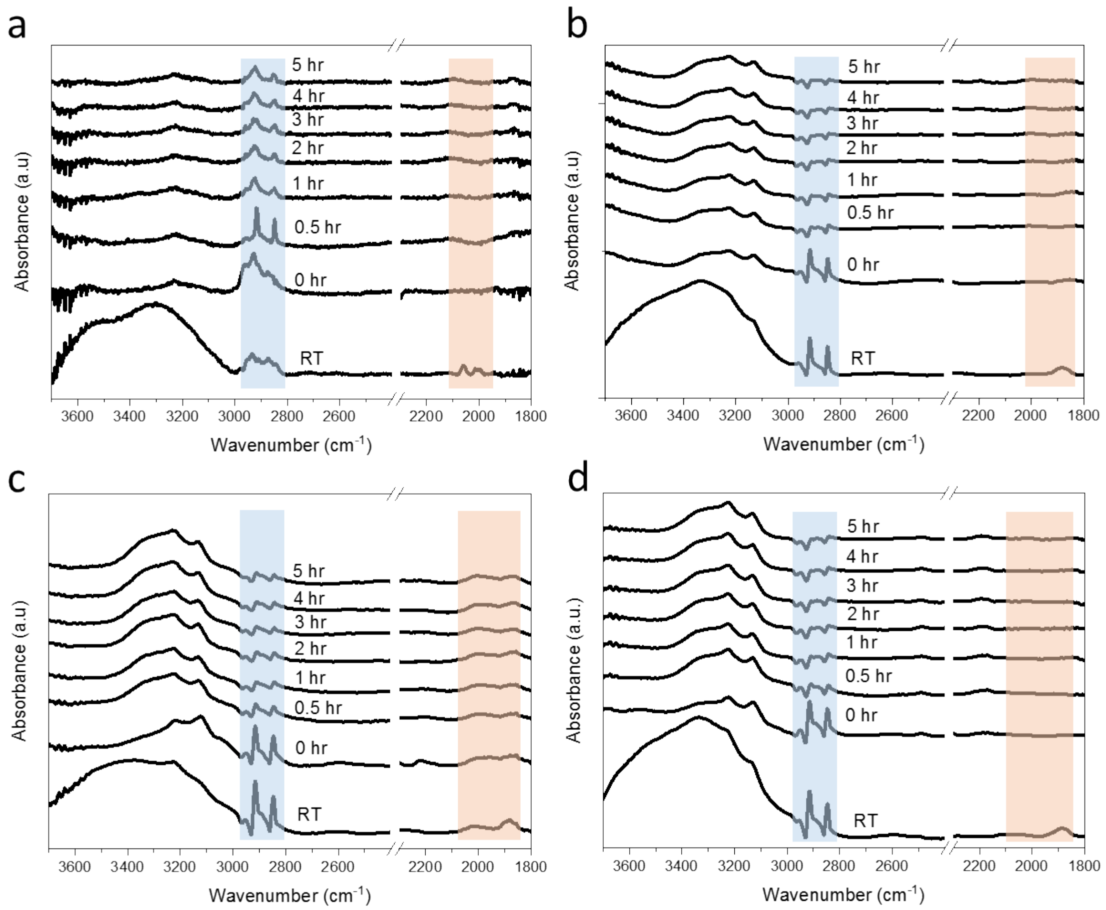

Figure S37. DRIFT measurements under oxidizing conditions at 250 °C of grafted (**a**) CuRu, (**b**) CuFe, (**c**) CuMo, (**d**) CuW on KIT-6. The spectrum brakes at 2300-2400 cm^{-1} due to dominant CO_2 signal that originates from residues at the flow cell.

5. Reactivity measurements

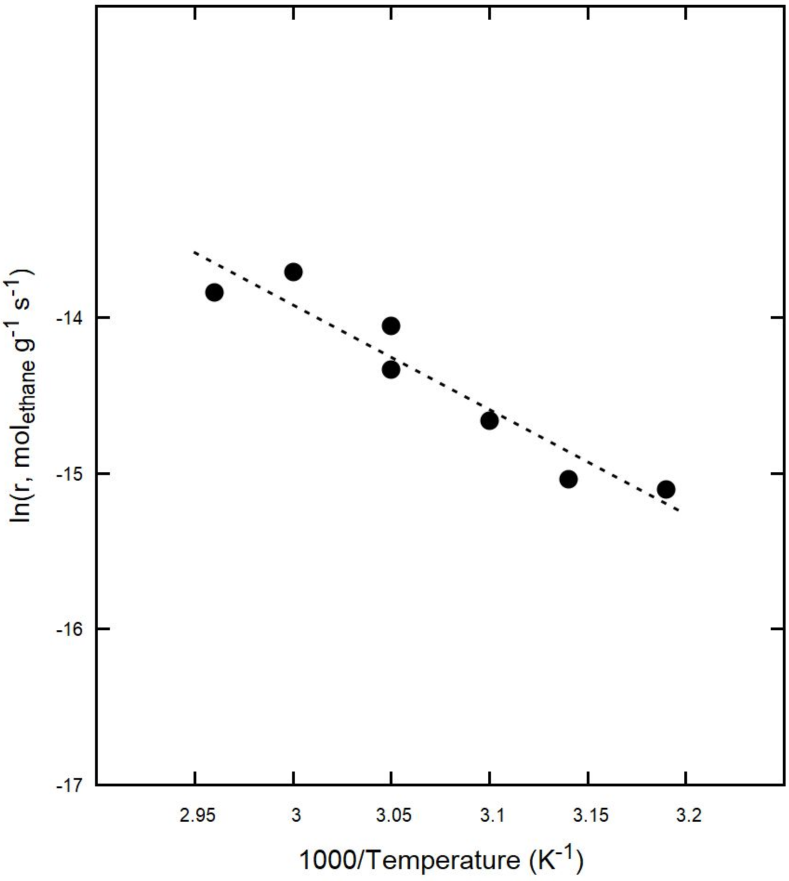

Figure S38. Reaction rate as a function of temperature for Cu nanoparticles on a silica support. Apparent activation energies were determined at 29.3 kPa hydrogen and 2.8 kPa ethylene at a total pressure of 111 kPa (balance helium). The gas hourly space velocity was held at 96 L g^{-1} catalyst h^{-1}.

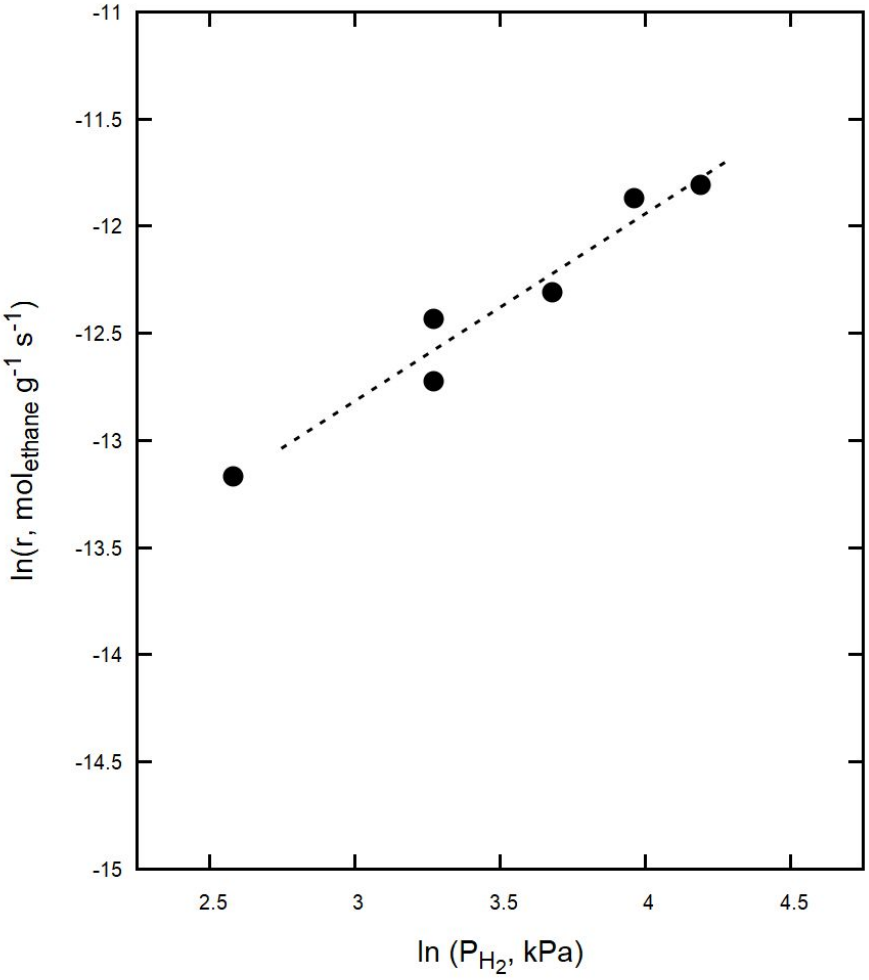

Figure S39. Reaction rate as a function of hydrogen pressure for Cu nanoparticles on a silica support. The reaction order was 0.87 at 75 °C. Ethylene pressure was held constant at 2.8 kPa while hydrogen pressure was varied over 14-73 kPa. The gas hourly space velocity was held at 96 L g^{-1} catalyst h^{-1}.

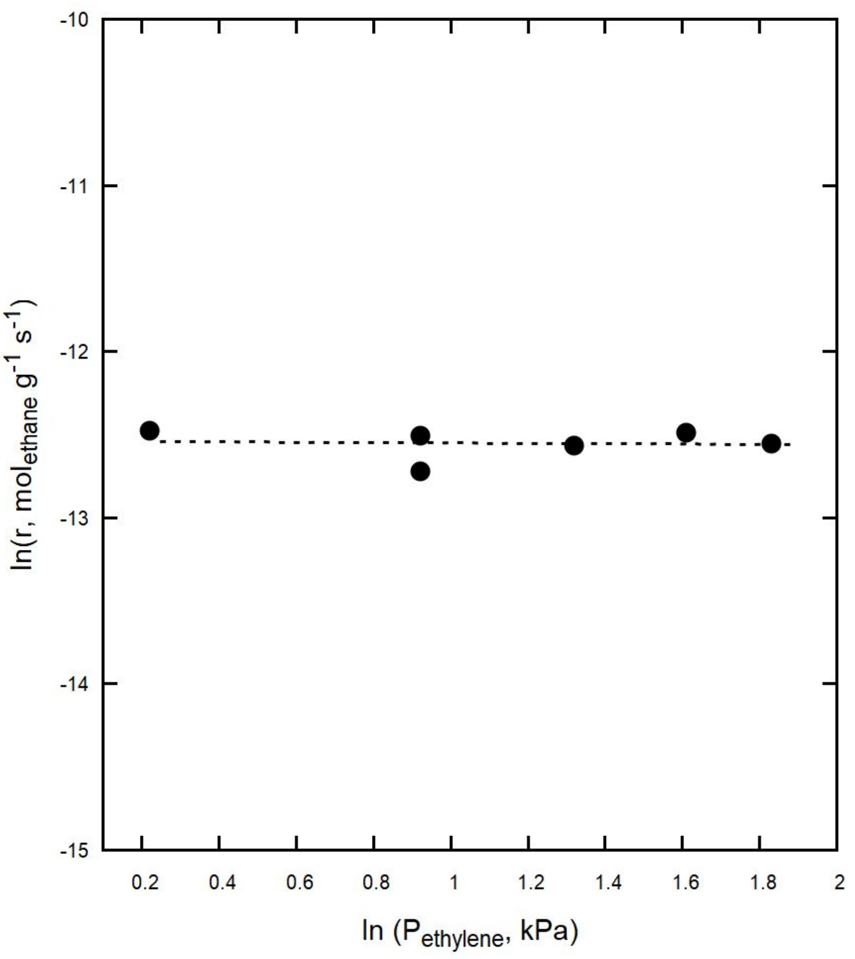

Figure S40. Reaction rate as a function of ethylene pressure for Cu nanoparticles on a silica support. The reaction order was -0.01 at 75 °C. Hydrogen partial pressure was held at 29.3 kPa while ethylene pressure was varied over 1.4-7.0 kPa. The gas hourly space velocity was held at 96 L g^{-1} catalyst h^{-1}.

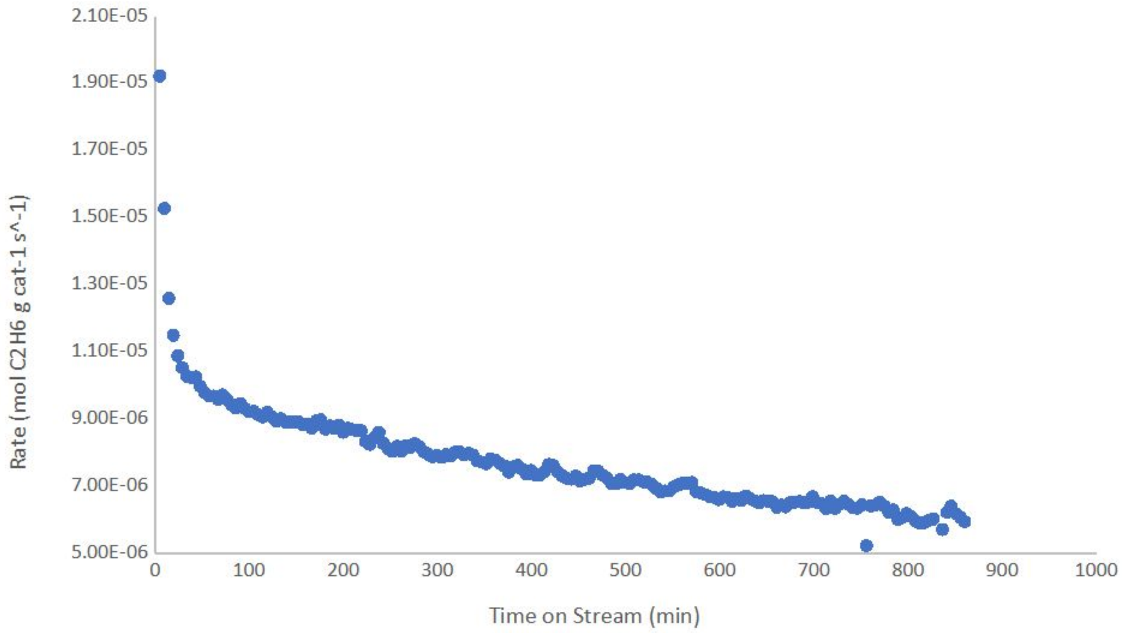

Figure S41. Long term reactivity analysis of CuRu catalyst.

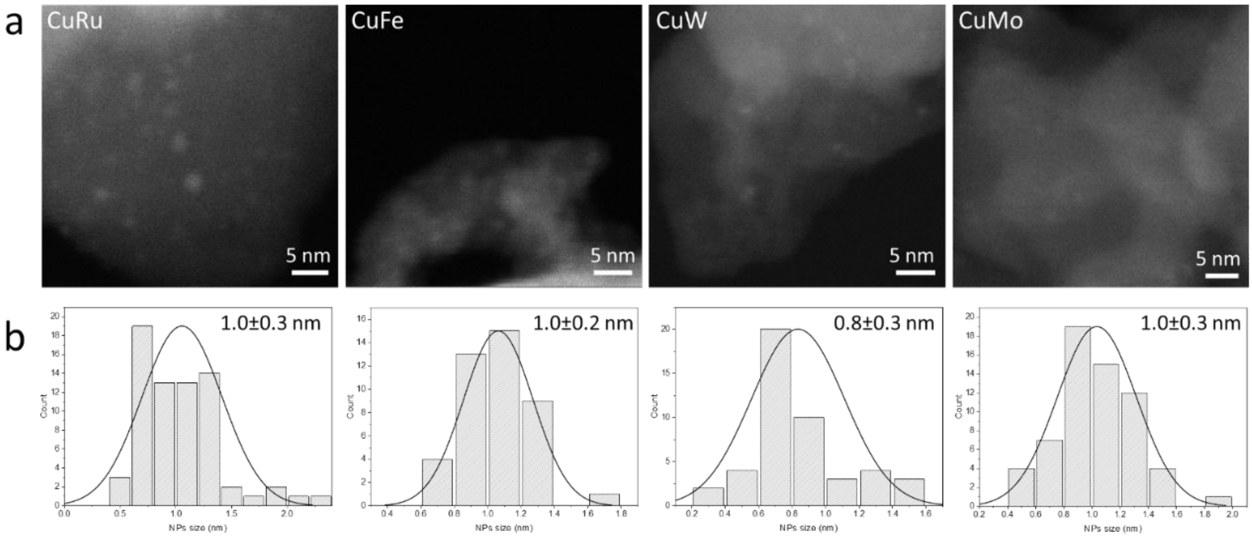

Figure S42. (**a**) HAADF-STEM images of Cu-M clusters after exposure to ethylene hydrogenation reaction conditions. (**b**) The corresponding size distribution analysis of the clusters shown in **a**.

6. References

1. Messervy, D. T.; Hayes, K. E., Kinetics of Copper-Catalyzed Hydrogenation of Ethylene. *Can J Chemistry* **1967**, *45* (6), 629-&.
2. Heard, C. J.; Hu, C. Q.; Skoglundh, M.; Creaser, D.; Grönbeck, H., Kinetic Regimes in Ethylene Hydrogenation over Transition-Metal Surfaces. *Acs Catal* **2016**, *6* (5), 3277-3286.
3. Wang, L. P.; Tysoe, W. T., An Investigation of Ethylene Hydrogenation Catalyzed by Metallic Molybdenum Using an Isolatable High-Pressure Reactor - Identification of the Reaction Site and the Role of Carbonaceous Deposits. *J Catal* **1991,** *128* (2), 320-336.
4. Emmett, P.H.; Gray, G.B., The hydrogenation of Ethylene, propylene, and 2-butene on Iron Catalysts. *JACS* **1944**, *66* (8) 1338-1343.
